# Isolation, Culture and Characterization of *Hirsutella sinensis* Mycelium from Caterpillar Fungus Fruiting Body

**DOI:** 10.1371/journal.pone.0168734

**Published:** 2017-01-03

**Authors:** Yun-Fei Ko, Jian-Ching Liau, Chien-Sheng Lee, Chen-Yaw Chiu, Jan Martel, Chuan-Sheng Lin, Shun-Fu Tseng, David M. Ojcius, Chia-Chen Lu, Hsin-Chih Lai, John D. Young

**Affiliations:** 1 Chang Gung Biotechnology Corporation, Taipei, Taiwan, Republic of China; 2 Biochemical Engineering Research Center, Ming Chi University of Technology, New Taipei City, Taiwan, Republic of China; 3 Chang Gung Immunology Consortium, Linkou Chang Gung Memorial Hospital, New Taipei City, Taiwan, Republic of China, and Chang Gung University, Gueishan, Taoyuan, Taiwan, Republic of China; 4 Center for Molecular and Clinical Immunology, Chang Gung University, Taoyuan, Taiwan, Republic of China; 5 Laboratory of Nanomaterials, Chang Gung University, Gueishan, Taoyuan, Taiwan, Republic of China; 6 Research Center of Bacterial Pathogenesis, Chang Gung University, Taoyuan, Taiwan, Republic of China; 7 Department of Medical Biotechnology and Laboratory Science, College of Medicine, Chang Gung University, Taoyuan, Taiwan, Republic of China; 8 Department of Microbiology and Immunology, College of Medicine, Chang Gung University, Taoyuan, Taiwan, Republic of China; 9 Department of Biomedical Sciences, University of the Pacific, Arthur Dugoni School of Dentistry, San Francisco, California, United States of America; 10 Department of Respiratory Therapy, Fu Jen Catholic University, New Taipei City, Taiwan, Republic of China; 11 Department of Laboratory Medicine, Linkou Chang Gung Memorial Hospital, New Taipei City, Taiwan, Republic of China; 12 Research Center for Industry of Human Ecology, College of Human Ecology, Chang Gung University of Science and Technology, Taoyuan, Taiwan, Republic of China; 13 Graduate Institute of Health Industry and Technology, College of Human Ecology, Chang Gung University of Science and Technology, Taoyuan, Taiwan, Republic of China; 14 Laboratory of Cellular Physiology and Immunology, The Rockefeller University, New York, New York, United States of America; University of California, Merced, UNITED STATES

## Abstract

The caterpillar fungus *Ophiocordyceps sinensis* (previously called *Cordyceps sinensis*) has been used for centuries in Asia as a tonic to improve health and longevity. Recent studies show that *O*. *sinensis* produces a wide range of biological effects on cells, laboratory animals and humans, including anti-fatigue, anti-infection, anti-inflammatory, antioxidant, and anti-tumor activities. In view of the rarity of *O*. *sinensis* fruiting bodies in nature, cultivation of its anamorph mycelium represents a useful alternative for large-scale production. However, *O*. *sinensis* fruiting bodies harvested in nature harbor several fungal contaminants, a phenomenon that led to the isolation and characterization of a large number of incorrect mycelium strains. We report here the isolation of a mycelium from a fruiting body of *O*. *sinensis* and we identify the isolate as *O*. *sinensis*’ anamorph (also called *Hirsutella sinensis*) based on multi-locus sequence typing of several fungal genes (ITS, nrSSU, nrLSU, RPB1, RPB2, MCM7, β-tubulin, TEF-1α, and ATP6). The main characteristics of the isolated mycelium, including its optimal growth at low temperature (16°C) and its biochemical composition, are similar to that of *O*. *sinensis* fruiting bodies, indicating that the mycelium strain characterized here may be used as a substitute for the rare and expensive *O*. *sinensis* fruiting bodies found in nature.

## Introduction

*Ophiocordyceps sinensis*—previously called *Cordyceps sinensis*—is an Ascomycetes fungus that grows at high altitude (3,500–5,000 m) and low temperature (~16°C) on the cold highlands of the Himalayas and the Qinghai-Tibetan plateau [1,2]. This fungus is known for its unusual parasitic life cycle: in late autumn, spores infect larvae of Hepialidae ghost moths in the soil, producing a mycelium that gradually consumes the insect’s internal organs; in summer, the mycelium forms a fruiting body that protrudes from the head of the dead insect and grows above the ground, facilitating spore dispersal and reproduction. *O*. *sinensis* is thus known in English as the “caterpillar fungus” while in Chinese it is called “winter-worm, summer-grass,” a name which reflects the unique life cycle of this organism.

*O*. *sinensis* has long been known in Asia as a popular folk remedy used to treat various ailments, including cancer, fatigue, impotence, liver disease, renal dysfunction, respiratory disease, and type 2 diabetes [3,4]. Recent studies have shown that *O*. *sinensis* produces a wide range of biological effects on cultured cells and laboratory animals, including anti-aging, anti-bacterial, anti-cancer, anti-diabetic, anti-fatigue, anti-inflammatory, anti-viral, immuno-modulatory, and lipid-lowering properties [2,5,6]. In humans, *O*. *sinensis* improves renal, hepatic and respiratory functions, delays fatigue, and reduces type 2 diabetes symptoms [3,4]. The fungus has also been used as an aphrodisiac, earning it the nickname “Himalayan Viagra” [1]. *O*. *sinensis* attracted international attention in 1993 when Chinese women athletes participating at the National Games in Beijing broke several world records at a single distance running event, performances which were later attributed (at least in part) to consumption of a tonic containing the caterpillar fungus [7]. For these reasons, the fungus has emerged as a major health supplement and tonic in recent years.

The high demand for *O*. *sinensis* fruiting bodies—especially in China but also throughout Asia—and the low annual production have led to overharvesting, a sharp production decline, as well as hefty price increases on the market (e.g., top-grade fruiting bodies were sold at $60,000/kg in 2007) [1]. *O*. *sinensis* has thus been listed as an endangered species in China [8,9]. Unfortunately, artificial cultivation of *O*. *sinensis* fruiting bodies on a large scale has continually failed [1], possibly due to the long life cycle of ghost moth insects and the absence of environmental cues needed to induce the mushroom’s fruiting process [10]. Moreover, *O*. *sinensis* fruiting bodies harvested in nature have been shown to contain relatively high levels of lead, arsenic, and copper [11], leading to cases of heavy metal poisoning [12]. Some *O*. *sinensis* fruiting bodies sold on the market are also adulterated with metal in order to increase product weight and sales profits [2]. For these reasons, much effort has been devoted to finding an alternative that is free of contaminants and that is amenable to large-scale culture under controlled laboratory conditions.

Several fungal species have been isolated from *O*. *sinensis* fruiting bodies collected in nature [13,14]. This phenomenon has led to the production and sale of several incorrect strains on the market [8,9]. For instance, the CS-4 strain sold in China has been shown to consist of *Paecilomyces hepiali* mycelium, while other fungi such as *Cordyceps militaris* fruiting bodies have been used as culture alternatives due to ease of production [8,9]. Confusion about the material described as *O*. *sinensis* is also rampant in the scientific literature, with a recent study estimating that more than three quarter of studies published on this fungus used unreliable, uncertain, or unspecified material [15]. Several fungal species have been proposed to represent the anamorph mycelium of *O*. *sinensis* [16], with some authors advocating that *Hirsutella sinensis* represents the sole anamorph [17–19], a claim that has been challenged by others [20]. Arguably no other fungus has created the level of attention and controversy seen here as far as culture, identification or characterization is concerned.

In the present study, we report the isolation of a mycelium from fresh *O*. *sinensis* fruiting body obtained in Tibet. Identification of the mycelium species was based on multi-locus sequence typing (MLST) of several fungal genes (ITS, nrLSU, nrSSU, RPB1, RPB2, MCM7, β-tubulin, EF-1α, and ATP6) [21,22], a strategy which to our knowledge has not been used previously to validate the isolation of *O*. *sinensis*’ anamorph. We show that the isolated mycelium strain closely matches the characteristics of wild *O*. *sinensis* fruiting bodies in terms of DNA sequences, culture conditions, and biochemical composition. The isolated mycelium thus represents a useful alternative for the production of health supplements containing the caterpillar fungus.

## Methods

### Strain isolation

Fresh *O*. *sinensis* fruiting bodies were purchased from a local vendor in the Naqu prefecture of Tibet in August 1999, a time when the organism had not yet been listed as an endangered species [9]. Therefore, no specific permissions were required in this case. For the isolation of *O*. *sinensis* (*H*. *sinensis*) strain CGB 999335, a fresh *O*. *sinensis* fruiting body (stroma section; 0.6–0.8 g) was briefly washed with sterile water, prior to immersion in 1% sodium hypochlorite (NaClO) for 1 min. Following subsequent wash with sterile water, the fruiting body was cut into small pieces (2–5 mm long) with a sterile scalpel and the pieces were placed in a bottle containing 5 ml of sterile water. The solution was homogenized with a blender prior to dilution 10 to 100× in sterile water. A small aliquot (100 μl) of the diluted solution was inoculated onto potato dextrose agar (PDA; 4 g/l potato extract, 20 g/l dextrose; 20 g/l agar) and cultured aseptically at various temperatures (10–35°C) for several days. Cultures were observed periodically and filamentous fungal colonies were selected and re-inoculated at least five times onto PDA plates to remove possible contaminants. A colony was selected and cultured in potato dextrose broth (PDB; same composition as PDA but without agar) at 18°C with gentle mixing. Stock culture was maintained at –80°C in 10% glycerol (v/v).

### Culture of *H*. *sinensis* mycelium

Mycelium colonies were cultured in FM1 liquid culture medium (20 g/l dextrose, 12 g/l yeast extract, 0.5 g/l K_2_HPO_4_, 0.25 g/l MgSO_4_•7 H_2_O, 0.05g/l FeSO_4_•7 H_2_O) or 1–10% (w/v) soybean broth at 18°C with gentle mixing for several days. Culture was performed at various temperatures (12–22°C). In some experiments, pH was adjusted (pH 4.2–8.0) with 1 M HCl or NaOH prior to culture. Mycelium cells were harvested by centrifugation at 3,400×g for 10 min using an Allegra 25R centrifuge (Beckman Coulter, Brea, CA). Mycelium cells were washed twice with double distilled water, prior to drying in an oven at 105°C. Dried mycelium powder was weighed to determine the amount of biomass following culture.

### Microscopy analysis

Mycelium cells from fresh liquid culture were visualized with an Olympus IX70 inverted optical microscope (Tokyo, Japan) equipped with a dark-field condenser; a Nikon Eclipse 80i upright optical microscope (Tokyo, Japan); or a Nikon SMZ1500 stereoscopic zoom microscope. Photography was taken with Nikon D100 and E995 digital cameras.

### PCR and DNA sequencing

Total genomic DNA was extracted from mycelium using glass beads as described before [22]. PCR amplification and sequencing of ITS1-5.8S-ITS2 rDNA, nrSSU, nrLSU, RPB1, RPB2 and MCM7 amplicons were conducted as previously described [21]. Amplification of EF-1α, β-tubulin and mtATP6 was performed based on established protocols [22]. The primers used are listed in S1 Table. PCR products were sequenced by Genomics BioSci & Tech (Taipei, Taiwan).

### Sequence alignment and phylogenetic analysis

BLASTN was used to identify sequences of highest homology (NCBI, Bethesda, MD). Sequences including Ophiocordycipitaceae, Bionectriaceae, Hypocreaceae, and Nectriaceae sensu lato and related species were obtained for 5-gene-based MLST (S2 Table; nrSSU, nrLSU, RPB1, RPB2, and EF-1α) and single-gene-based phylogenetic tree analysis (S3 Table). Raw sequences were aligned and gaps were excluded using ClustalW. The Molecular Evolutionary Genetics Analysis software (MEGA, version 6.06) was used to perform phylogenetic analysis. For 5-gene-based MLST analysis, evolutionary history was inferred using the maximum composite likelihood (MCL) method based on the Tamura-Nei model and phylogenetic tree of the heuristic search was obtained using the neighbor-joining and BioNJ algorithms to obtain a matrix of pairwise distances estimated using the MCL approach inferred from 500 bootstrap replicates [23–25]. For single gene-based phylogenetic analysis, the evolutionary history was inferred using the neighbor-joining method [26] combined with the MCL-based evolutionary distance estimation [24]. The percentage of replicate trees in which the associated taxa clustered together is shown next to the branches of the bootstrap consensus tree as before [27]. Novel DNA sequences of *O*. *sinensis* (*H*. *sinensis*) CGB 999335 were deposited in the NCBI database (KU058601, KU239984–KU239991).

### Energy and chemical analysis

Determination of the content of organic compounds and elements in dried HSM CGB999335 mycelium and *O*. *sinensis* fruiting bodies was performed by SGS Taiwan (New Taipei City, Taiwan) using standard procedures.

### High-performance liquid chromatography analysis

The high-performance liquid chromatography (HPLC) system (Waters, Milford, MA) consisted of a series 600 controller, a series 717 plus autosampler, and a series 996 photodiode-array detector, connected to a cartridge column (GL Sciences, Tokyo, Japan; average particle size of 5 μm) and a Cosmosil packed 5C_18_-MS-II column (Nacalai, San Diego, CA; internal diameter of 4.6×250 mm; average particle size of 5 μm). The mobile phase consisted of buffer A (2.5% methanol in 0.01 M ammonium dihydrogen phosphate, pH 5.3) and buffer B (20% methanol in 0.01 M ammonium dihydrogen phosphate, pH 5.1). Elution started with 100% buffer A and consisted of the following linear gradient steps: 0–10 min, 0–25% buffer B; 10–20 min, 25–40% buffer B; 20–60 min, 40–100% buffer B. A flow rate of 0.9 ml/min and an injection volume of 20 μl was used. Temperature of the column was maintained at 25°C. Detection was done at a wavelength of 260 nm. Deionised water used for preparation of the HPLC mobile phase and sample dilution was prepared with the Milli-Q purification system (Millipore, Bedford, MA). Nitrogenous bases, nucleosides, HPLC-grade methanol, and ammonium dihydrogen phosphate were obtained from Sigma-Aldrich (St. Louis, MO).

### Statistical analysis

Experiments were performed in triplicate. Results are expressed as means ± standard errors (SE). Statistical significance was evaluated using Student’s *t*-test and a significance threshold of 5%.

## Results

### Characteristics of the mycelium isolated from *O*. *sinensis* fruiting body

**Fig 1:**
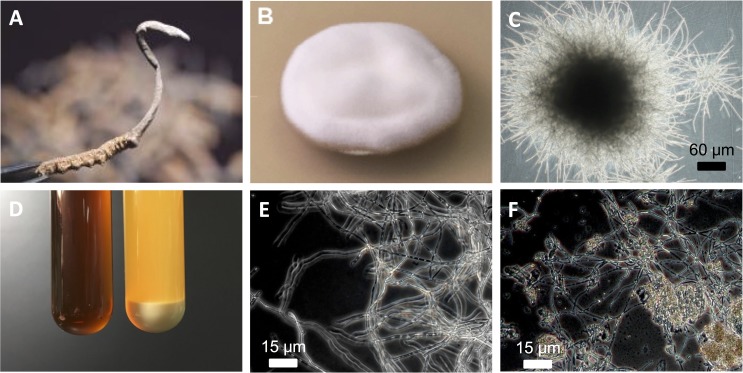
Culture of *H*. *sinensis* mycelium derived from *O*. *sinensis* fruiting body. (A) *O*. *sinensis* fruiting body or stroma (top) protruding from the shell of a caterpillar insect (bottom) was obtained in the Naqu prefecture in Tibet. HSM strain CGB 999335 was isolated from a similar fruiting body. (B) Colony of HSM strain CGB 999335 cultured for 28 days at 18°C on PDA agar. (C) CGB 999335 mycelium observed under optical microscopy. (D) Sterile FM1 liquid medium used to culture CGB 999335 mycelium in the present study (left tube; containing 1.2% of yeast extract as a source of nitrogen) and sterile liquid 1.2% (w/v) soybean broth commonly used in other laboratories (tube on the right). Notice the pellet of undissolved powder in the tube on the right. See the Methods section for more details. (E) Dark-field optical microscopy image of CGB 999335 mycelium cultured in FM1 medium. (F) CGB 999335 mycelium cultured in soybean broth seen in D (tube on the right). Undissolved, brown material can be seen among mycelial cells.

Given the repeated isolation of fungal contaminants from *O*. *sinensis* fruiting bodies [13] and the controversy surrounding the identification of *O*. *sinensis* and its anamorph [8,9,16], we aimed to culture a mycelium from fresh fruiting bodies of *O*. *sinensis* (obtained in Naqu Prefecture, Tibet). The *O*. *sinensis* specimen consisted of the characteristic caterpillar shell from which a fruiting body of *O*. *sinensis* had protruded (an example of a dried specimen is shown in Fig 1A). After gentle wash, the stroma of the fruiting body was cut into small pieces and incubated onto potato dextrose agar (PDA) at low temperature (10–20°C), in order to mimic the low temperature at which *O*. *sinensis* grows in the wild. After serial passages of single colonies, we isolated a mycelium that produced diffuse, white colonies with dense aerial mycelium and regular margins on PDA medium (Fig 1B, 28 days of culture). Under optical microscopy, we observed that mycelium cells were hyaline, branched and smooth-walled (Fig 1C).

We cultivated the mycelium in FM1 liquid medium, which is entirely soluble and free of debris (Fig 1D, left tube). Optical microscopy images of HSM mycelium cultured in FM1 showed abundant interlaced, branched, and hyaline mycelium cells with thin cell walls and intercellular septa (Fig 1E). In contrast, when the mycelium was cultured in soybean broth (Fig 1D, tube on the right)—a medium commonly used to culture *Cordyceps*-related mycelium [28–30]—undissolved material and culture debris were observed among mycelium cells (Fig 1F).

### Identification of *H*. *sinensis* using multi-locus sequence typing

**Fig 2:**
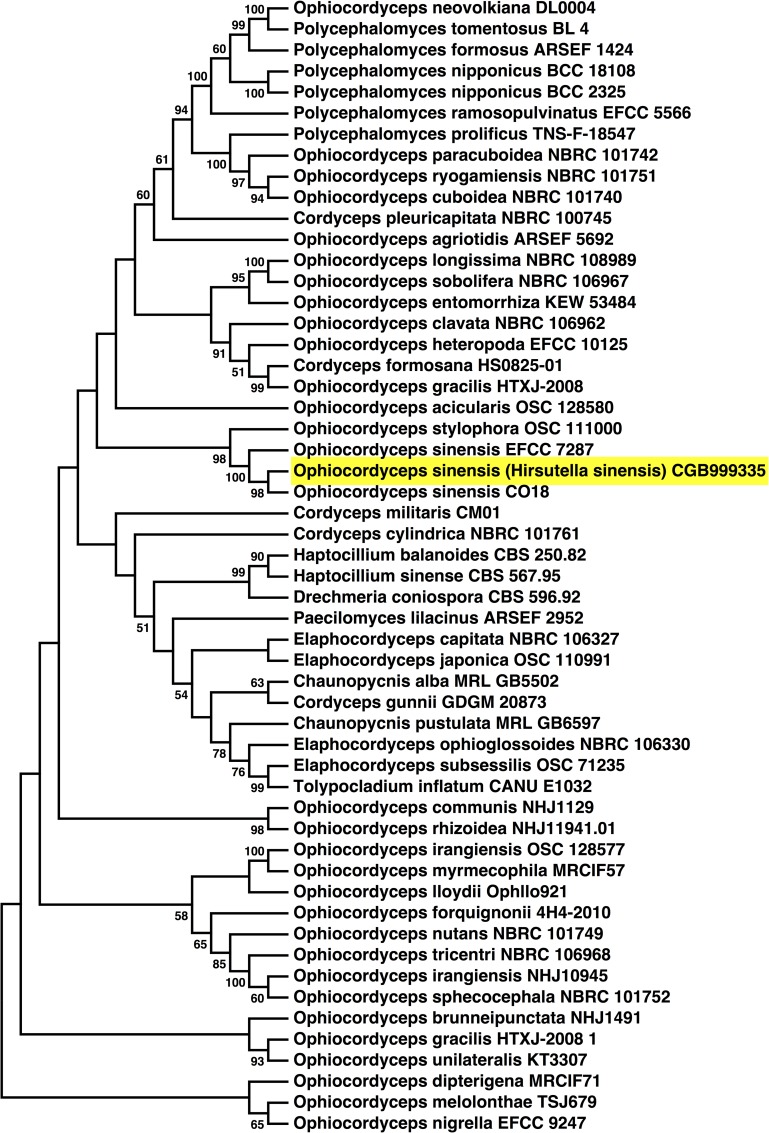
5.8S-ITS rDNA phylogenetic tree of *Ophiocordyceps* species. The evolutionary relationship of *Ophiocordyceps* 5.8S-ITS rDNA genes was determined using the neighbor-joining method [32]. Evolutionary distances were assessed using the maximum composite likelihood (MCL) method. The bootstrap consensus tree, which represents the evolutionary relationship of the analyzed taxa, was inferred from 500 replicates as before [27]. Branches corresponding to partitions reproduced in less than 50% bootstrap replicates are collapsed. The percentage of replicate trees in which the associated taxa clustered together in the bootstrap test (500 replicates) is shown next to the branches. Evolutionary distances (i.e., number of base substitutions per site) were computed using the maximum composite likelihood method [24].

To identify the mycelium species, we amplified internal transcribed spacer regions 1 and 2 (ITS1 and ITS2) and 5.8S rRNA by PCR and sequenced the obtained amplicons, a technique used in the past to identify fungal species [21,22]. BLASTN search revealed that the best sequence match was *O*. *sinensis* strain HMAS:173825 (S1 Fig; 100% identity). The neighbor-joining statistical method was used to build a phylogenetic tree and to compare the 62 ITS-5.8S-rRNA sequences with the highest level of homology (Fig 2). While recent nomenclature guidelines encourage the use of “one fungus, one name” [31], we refer to the isolated mycelium as *O*. *sinensis* (*H*. *sinensis* mycelium) HSM strain CGB999335, or in short HSM CGB999335, in order to provide additional information about the strain. We found that HSM CGB999335 clustered with other *O*. *sinensis* strains (Fig 2, EFCC 7287 and CO18), suggesting a common origin.

We further confirmed the identity of HSM CGB999335 by amplifying and sequencing eight additional housekeeping genes used for the identification of fungal species [21,22,33]. The genes sequenced included the small and large 18S nuclear ribosomal RNA subunits (nrSSU and nrLSU), the largest and second largest subunits of RNA polymerase II (RPB1 and RPB2), mini-chromosome maintenance complex component 7 (MCM7), β-tubulin, translation elongation factor 1-alpha (TEF1-α), and mitochondria ATPase synthase subunit 6 (ATP6). Results were analyzed using BLASTN and the neighbor-joining statistical method (see Methods).

Based on the genes analyzed, HSM CGB999335 showed a high level of homology to other *O*. *sinensis* isolates deposited in the database (S1–S17 Figs). Notably, HSM CGB999335 showed high homology to *O*. *sinensis* isolate CO18 (S7–S17 Figs), a legitimate caterpillar fungus strain isolated on the Qinghai-Tibetan plateau and whose genome has been partially sequenced (GenBank: KE659607.1) [10]. As shown in S1–S4, S7 and S8 Figs, HSM CGB999335 showed 100% identity to several *O*. *sinensis* isolates for several genes, including ITS-5.8S-rRNA (strain HMAS:173825), nrSSU (strain SJL0809), nrLSU (strain SJL0809), RPB1 (strain EFCC 7287), MCM7 (strain CO18), and β-tubulin (strain CO18). High identity scores were also obtained for RPB2 (S5 Fig, 99.7% identity) and TEF1-α (S6 Fig, 99.8% identity) of *O*. *sinensis* isolate YN07-8, as well as for ATP6 of *O*. *sinensis* isolate CO18 (S9 Fig, 99% identity). Phylogenetic trees showing the evolutionary relationships between HSM CGB999335 and related species for nrSSU, nrLSU, RPB1, RPB2, TEF-1α, MCM7, β-tubulin and ATP6 are shown in S10–S17 Figs.

**Fig 3:**
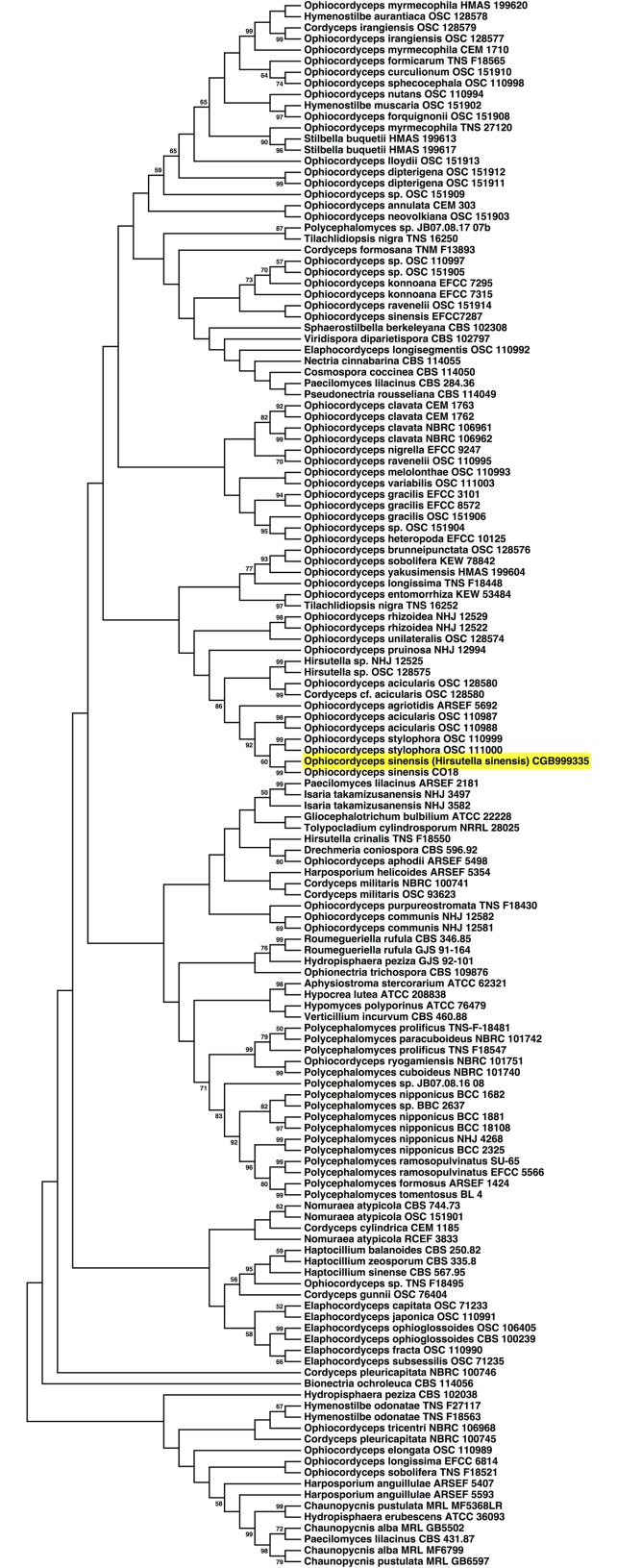
Multi-locus sequence typing-based phylogenetic analysis of *Ophiocordycipitaceae*, *Bionectriaceae*, *Hypocreaceae* and *Nectriaceae* for a five-gene dataset. The evolutionary relationship of a five-gene dataset (nrSSU, nrLSU, RPB1, RPB2, EF-1a) was determined using the maximum likelihood method based on the Tamura-Nei model [25]. Initial tree(s) for the heuristic search were obtained using the neighbor-joining and BioNJ algorithms to a matrix of pairwise distances estimated using the maximum composite likelihood (MCL) approach. Shown here is the bootstrap consensus tree inferred from 500 bootstrap replicates. The percentage of replicate tree that clustered with associated taxa is indicated.

A phylogenetic tree based on a five-gene dataset (nrSSU, nrLSU, RPB1, RPB2, EF-1a) and the 141 most homologous species showed that HSM CGB999335 has the highest homology to *O*. *sinensis* strain CO18 (Fig 3). Based on these results, we conclude that the isolated HSM CGB999335 mycelium is most related to *O*. *sinensis* fruiting bodies harvested in nature. These results are in agreement with previous studies showing that the anamorph of *O*. *sinensis* corresponds to *H*. *sinensis* [17,18].

### Optimal culture conditions of *O*. *sinensis* mycelium

**Fig 4:**
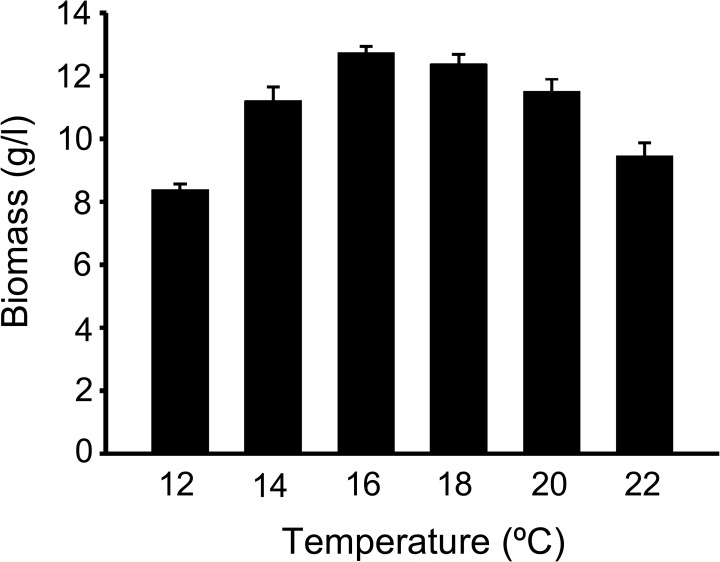
Effect of temperature on the culture of *H*. *sinensis* mycelium. CGB 999335 mycelium was cultured in liquid FM1 medium with mixing for eight days at the temperature indicated. Mycelium cells were obtained by centrifugation, followed by drying and measurement of biomass weight.

To analyze the growth characteristics of the HSM CGB999335 strain, we established a liquid culture using the liquid FM1 medium. This allowed us to precisely monitor the growth characteristics of the mycelium in addition to producing a sufficient amount of biomass for subsequent analysis. We cultured HSM CGB999335 at different temperatures from 12 to 24°C in order to determine the temperature that produces optimal growth. HSM CGB999335 produced optimal growth at 16°C (Fig 4), consistent with previous observations that *O*. *sinensis* mycelium grows best at 15 and 18°C [15]. HSM biomass gradually decreased at temperatures above 16°C and limited growth was noticed at higher temperatures, consistent with previous work showing that *O*. *sinensis* does not grow at temperatures above 25°C [15].

**Fig 5:**
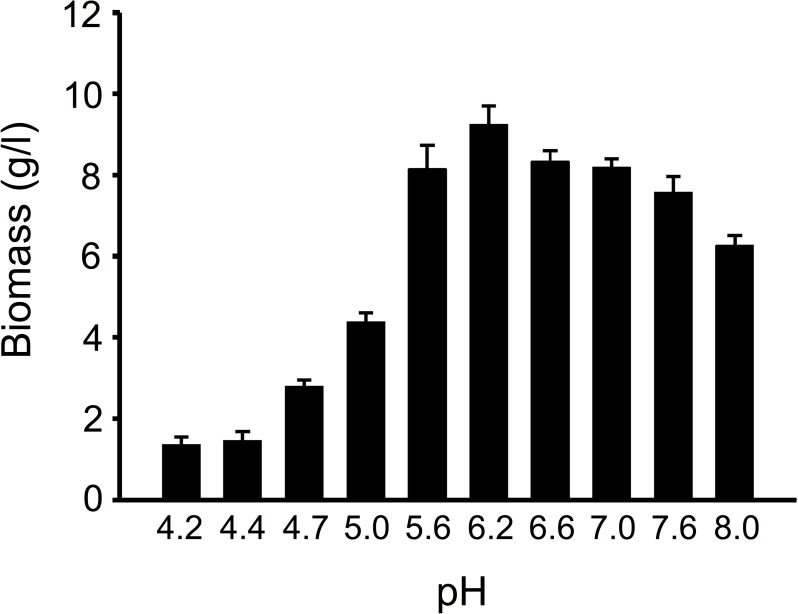
Culture of *H*. *sinensis* mycelium at various pH. CGB 999335 mycelium was cultured in liquid FM1 medium at 16°C with mixing. Prior to culture, the pH of the culture medium was adjusted to the indicated value by adding 1 M HCl or NaOH. After five days of culture, mycelium cells were obtained by centrifugation, followed by drying and measurement of biomass weight.

To determine whether pH has any effect on mycelium growth, we cultured HSM in FM1 liquid culture media at various pH, ranging between 4.2 to 8.0. After five days of culture, we observed that the culture medium with a pH of 6.2 produced the highest amount of mycelium biomass (Fig 5). These results are in agreement with previous observations that *O*. *sinensis*-derived mycelium grows best at pH 6 [15].

**Fig 6:**
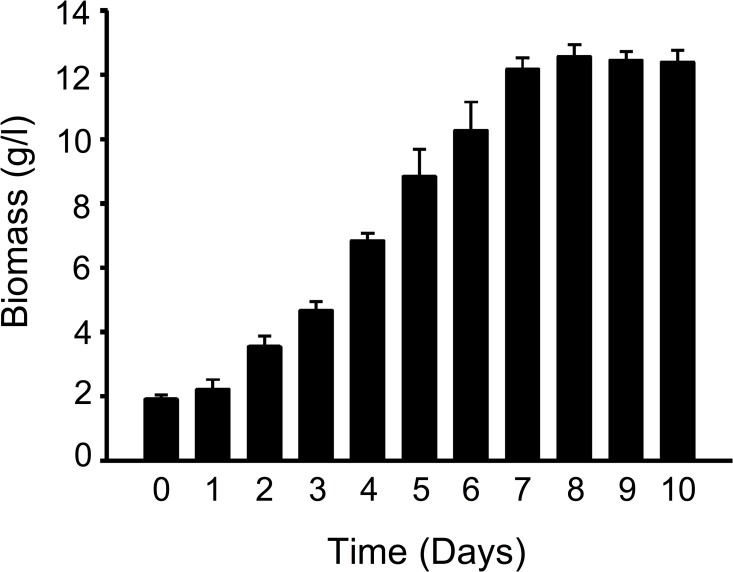
Culture of *H*. *sinensis* mycelium with time. CGB 999335 mycelium was cultured in liquid FM1 medium with mixing at 16°C for the time indicated. Mycelium cells were obtained by centrifugation, followed by drying and measurement of biomass weight.

We also analyzed the amount of biomass produced after several days in culture. Using a temperature of 16°C and pH 6.2, we observed that a culture time of 8 days produced the highest amount of mycelium biomass (Fig 6). No further increase or decline of biomass was noted after this period (Fig 6). Taken together, these results suggest that the HSM CGB999335 strain possesses growth characteristics similar to that of *O*. *sinensis* mycelium characterized in previous studies and the fruiting body harvested in the wild. Culture conditions may thus be critical for the isolation of *O*. *sinensis* mycelium.

### Chemical analysis

In order to characterize the HSM CGB999335 strain isolated here, we compared its composition with that of *O*. *sinensis* fruiting bodies. The HSM strain showed higher levels of energy, proteins, lipids, and superoxide dismutase compared with *O*. *sinensis* fruiting bodies (Table 1). On the other hand, HSM showed lower levels of carbohydrates and water than the fruiting bodies, while the amount of polysaccharides was similar in both samples (Table 1). HSM showed low levels of saturated fatty acids and sugars, while these molecules were not detected in *O*. *sinensis* fruiting bodies (Table 1). These observations suggest that the composition of HSM CGB999335 is comparable to that of *O*. *sinensis* fruiting bodies.

**Table 1:** Energy and chemical analysis of *H*. *sinensis* mycelium.

Component	CGB 999335 Mycelium (per 100 g)	*O*. *sinensis* Fruiting Body (per 100 g)
Energy	373.0 kcal	348.5 kcal
Proteins	42.8 g	30.1 g
Polysaccharides	3.8 g	4.0 g
Lipids	8.2 g	5.0 g
Saturated fatty acids	0.6 g	ND (<0.3 g)
Carbohydrates	31.9 g	45.8 g
Sugars	2.8 g	ND
Water	< 5 g	< 12 g
Superoxide dismutase	2.3 × 10^5^ U	1.5 × 10^5^ U

kcal, kilo-calorie; ND, not detected; U, unit.

**Fig 7:**
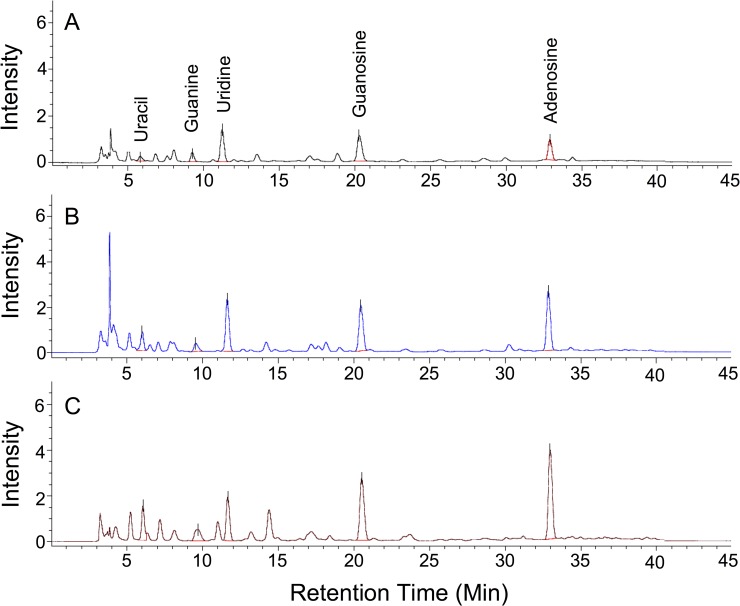
HPLC chromatograms of *H*. *sinensis* mycelium and natural *Ophiocordyceps* specimens. HPLC chromatogram of (A) *O*. *sinensis* fruiting body and insect; (B) *O*. *sinensis* fruiting body; and (C) CGB 999335 mycelium. HPLC was performed on a reverse-phase column as described in *Methods*. Peaks were identified based on the use of pure standard compounds processed under the same conditions. Nucleosides and nitrogenous bases were monitored using a UV detector. The results shown are representative of experiments performed in triplicate. Peak intensities are given in Table 2.

Nucsleosides have been described as major active compounds responsible for the biological effects of *O*. *sinensis* [5]. We therefore compared the content of nitrogenous bases and nucleosides in HSM CGB999335 and *O*. *sinensis* fruiting bodies by using high-performance liquid chromatography (HPLC). Pure nucleosides and nitrogenous bases were processed in parallel as positive controls. Chromatograms of *O*. *sinensis* fruiting bodies revealed the presence of uracil, guanine, uridine, guanosine, and adenosine (Fig 7B). Notably, the chromatogram of HSM CGB999335 showed highly similar peaks (Fig 7C vs. 7B), with minor variations in intensity. For comparison, we also processed *O*. *sinensis* fruiting bodies in complex with the moth insect (which is usually used to prepare TCM remedies); similar nucleosides and nitrogenous bases were found in this case as well, although peak intensities were relatively lower than for the fruiting bodies or HSM (Fig 7A vs 7B and 7C).

By measuring the area under the curves for each HPLC peak (Fig 7), we obtained a quantitative analysis of each nucleoside and nitrogenous base (Table 2). Except for uridine, HSM CGB999335 showed a higher content of nucleosides and nitrogenous bases compared with *O*. *sinensis* fruiting bodies (Table 2). By contrast, uracil was significantly higher in HSM CGB999335, up to 4.51 fold, in contrast with the *O*. *sinensis* fruiting bodies (Table 2). The level of adenosine in HSM CGB999335 was higher compared with the *O*. *sinensis* fruiting bodies (Table 2). Of note, cordycepin was not detected in any of the samples studied here. Based on these observations, we conclude that the nucleoside and nitrogenous base composition of HSM CGB999335 and *O*. *sinensis* fruiting bodies is highly similar.

**Table 2:** Peak intensity of nucleosides detected in *H*. *sinensis* as analyzed by HPLC.

	CGB 999335 Mycelium	*O*. *sinensis* Fruiting Body	*O*. *sinensis +* Insect
Uracil	1,993,190	1,192,030	326,990
Guanine	1,373,310	720,160	601,910
Uridine	3,014,090	3,743,940	2,309,260
Guanosine	5,369,850	3,873,900	2,374,820
Adenosine	7,001,920	4,686,090	1,515,800

Nucleoside peaks correspond to those shown in Fig 7.

Mushrooms grown in nature tend to assimilate various elements from the soil, including heavy metals [34,35]. We compared the elemental composition of HSM CGB999335 with that of *O*. *sinensis* fruiting bodies. We observed that HSM CGB999335 contains higher levels of potassium, zinc, calcium and sodium compared with *O*. *sinensis* fruiting bodies (Table 3). However, HSM harbors lower levels of magnesium, iron, and manganese (Table 3). Of note, HSM contains less than 10 ppm of heavy metals (e.g., lead, arsenic, mercury, cadmium, and copper; data not shown) and chromium and selenium were not detected in our samples (Table 3).

**Table 3:** Elemental analysis of *H*. *sinensis* mycelium.

Element	CGB 999335 Mycelium (ppm)	*O*. *sinensis* Fruiting Body (ppm)
Potassium (K)	14,188	8,058
Magnesium (Mg)	3,445	7,184
Zinc (Zn)	120	77
Iron (Fe)	35	1,972
Chromium (Cr)	ND	ND
Manganese (Mn)	16	60
Selenium (Se)	ND	ND
Calcium (Ca)	2,688	1,400
Sodium (Na)	243 mg/100 g	128 mg/100 g

ND: not detected.

We also analyzed the content of amino acids and other related organic compounds in HSM CGB999335 and *O*. *sinensis* fruiting bodies (Table 4). A total of 17 amino acids were detected in both samples, including the essential amino acids histidine, isoleucine, leucine, lysine, methionine, phenylalanine, threonine, tryptophan, and valine (Table 4). HSM contained higher levels of most of the compounds tested (n = 39), with the exceptions of seven compounds which were found at higher levels in *O*. *sinensis* fruiting bodies (Table 4; 2-aminoisobutyric acid, arginine, ethanolamine, ornithine, phenylalanine, phosphoethanolamine, and serine). Taken together, these observations indicate that the compositions of HSM CGB999335 and *O*. *sinensis* fruiting bodies are strikingly similar.

**Table 4:** Analysis of amino acids and selected organic compounds in *H*. *sinensis* mycelium.

Compound	CGB 999335 Mycelium (mg/100 g)	*O*. *sinensis* Fruiting Body (mg/100 g)
β-Alanine	31.8	25.7
L-Alanine	405.1	203.3
L-2-Aminoadipic acid	26.8	5.6
DL-2-Aminobutyric acid	25.2	11.3
γ-Aminobutyric acid	257.6	79.4
DL-2-Aminoisobutyric acid	20.0	29.4
L-Anserine	105.3	ND
L-Arginine	123.3	283.0
L-Asparagine	ND	ND
L-Aspartic acid	137.2	122.1
L-Carnosine	ND	ND
L-Citrulline	ND	ND
L-Cystathionine	57.4	25.4
L(–)-Cystine	ND	ND
Ethanolamine	18.4	38.6
L-Glutamic acid	800.1	530.9
L-Glycine	97.6	44.2
L-Histidine	100.4	99.8
DL-(+)-allo-δ-Hydroxylysine	15.2	8.5
L-Hydroxyproline	ND	ND
L-Isoleucine	113.5	34.0
L-Leucine	118.8	67.9
L-Lysine	254.7	200.1
L-Methionine	36.0	21.5
L-1-Methylhistidine	ND	ND
L-3-Methylhistidine	ND	ND
L-Ornithine	69.6	112.7
L-Phenylalanine	32.8	51.8
o-Phosphoethanolamine	71.5	99.3
o-Phosphoserine	ND	ND
L-(–)-Proline	254.4	90.9
Sarcosine	ND	ND
L-Serine	64.4	102.8
Taurine	74.4	47.0
L-Threonine	75.1	65.2
L-Tryptophan	28.7	4.9
L-Tyrosine	144.1	41.3
Urea	ND	ND
L-Valine	337.2	86.9

ND: not detected.

## Discussion

Identification of the mycelium anamorph of *O*. *sinensis* has been controversial, mainly due to contamination of fruiting bodies by various fungal species [13] and the difficulty in cultivating fruiting bodies in vitro [10]. A mycelium culture that can be used as an alternative for the declining production of *O*. *sinensis* fruiting bodies is highly needed. We report here the isolation of a mycelium from *O*. *sinensis* fruiting bodies harvested on the Qinghai-Tibetan plateau. Using a comprehensive PCR-based MLST analysis, we confirmed that the mycelium isolate corresponds to the anamorph of *O*. *sinensis* as shown by the high level of DNA sequence homology with *O*. *sinensis* sequences deposited in the NCBI database, including a legitimate *O*. *sinensis* strain isolated on the Qinghai-Tibetan plateau (i.e., CO18 [10]). To our knowledge, this work is the first independent isolation and characterization of the anamorph of *O*. *sinensis* outside of China that is based on in-depth DNA sequencing analysis. Notably, the HSM CGB999335 isolate shows growth characteristics and a biochemical composition similar to that of *O*. *sinensis* fruiting bodies found in the wild, further supporting our conclusion that this mycelium corresponds to the anamorph stage of the caterpillar fungus.

Previous studies performed on the HSM CGB999335 strain characterized here have shown that this strain produces several beneficial effects on cultured cells and laboratory animals. For instance, we observed earlier that an ethanol extract of the same HSM strain suppressed interleukin-1β secretion and inflammasome activation in human macrophages [36]. The ethanol extract also reduced bleomycin-induced lung injury, inflammation and fibrosis in mice [37], indicating that the extract may be used to treat conditions associated with chronic inflammation. Recently, we observed that a water extract of the HSM strain enhanced the cytotoxic activity of natural killer cells against cancer cells, whereas the ethanol extract of the strain reduced cytotoxicity [38]. These observations led us to propose that water and ethanol extracts of medicinal mushrooms may produce opposite effects on immune cells. In another study, Shang et al. showed that the HSM strain characterized here reduces the growth of hepatocellular carcinoma in nude mice [39]. Furthermore, Wu et al. showed that the same HSM strain reduces fatigue in mice as shown by increased time to exhaustion in swimming experiments [40]. Similar immunomodulatory, anti-cancer and anti-fatigue effects have been reported for *O*. *sinensis* fruiting bodies [3,4], further supporting the view that the HSM CGB999335 strain isolated here may be used as a substitute for the rare and expensive fruiting bodies found in nature.

We observed that HSM CGB999335 grows best at low temperature (16°C), which is similar to previous observations on the optimal growth of *O*. *sinensis* mycelium [15]. Previous studies claimed that *O*. *sinensis*-derived mycelium can be cultured at various temperatures ranging from 18 to 30°C (see the studies described in ref. [15]). For instance, mycelium characterized as *P*. *hepiali* [41] and *Tolypocladium* sp. [42] produced abundant growth at 25°C. On the other hand, we observed that growth temperatures of 15–20°C represent a limiting range of temperatures for the isolation of the mycelium anamorph of *O*. *sinensis*, a finding which is consistent with previous studies [17]. These results suggest that the conditions of isolation, especially the low temperature and the use of fresh fruiting bodies, may be crucial for the isolation of *O*. *sinensis* anamorph.

Culture of *O*. *sinensis*-derived mycelium for commercial purpose has usually been performed using liquid cell culture media containing corn extract, milk powder, silkworm pupa, soybean extract, wheat bran, or yeast extract [28–30,43–45]. When prepared at 1–10% (w/v) in water, these culture media may harbor a pellet of insoluble matter, even prior to culture of the fungus (see for instance the tube on the right in Fig 1D). Following culture of the mycelium for several days, the pellet of undissolved culture medium initially present in solution is only partially consumed by mycelium cells, a process that leaves corn, milk, silkworm, soybean, wheat or yeast residues in the final mycelium culture (Fig 1F). This strategy is likely to decrease not only the purity of the final mycelium extract but also the biological effects it produces on animals and humans. In addition, individuals who consume mycelium products cultured this way may develop allergies to culture medium residues, a phenomenon reported earlier for mycelium cultured in silkworm-containing media [46]. In contrast, we used a fully-soluble liquid culture medium (Fig 1D, left tube), a strategy that may favor maximal product yield, purity and efficacy, in addition to reducing the likelihood of allergic reactions.

Cordycepin has been proposed to represent a major active compound of *O*. *sinensis*. We did not detect this compound in any of the samples submitted to HPLC analysis (Fig 7), in spite of appropriate controls processed under the same conditions. This observation suggests that a reevaluation of the role of cordycepin in *O*. *sinensis* fruiting bodies and mycelium is needed. Species such as *C*. *militaris* contain cordycepin while *O*. *sinensis* fruiting bodies and cultured mycelium contained minor traces or insignificant amounts of the compound [47], suggesting the possibility that mislabeled *O*. *sinensis* samples may have been used in past studies in which cordycepin was detected at relatively high concentrations. Some authors suggested that nucleosides could be used to evaluate the quality of *Cordyceps* specimens [48]. Accordingly, adenosine may be used as a marker to evaluate the quality of the mycelium isolated and final *O*. *sinensis* products available on the market.

Several *O*. *sinensis*-related products have been commercialized on the market [8,9]. On the other hand, it appears unlikely that these products, which in some cases correspond to species different from *O*. *sinensis* or *H*. *sinensis*, all produce the same effects on laboratory animals and humans. The use of different species may be due to the fact that identification of *O*. *sinensis* is often based solely on morphological criteria or isolation of mycelium from *O*. *sinensis* fruiting bodies, in the absence of DNA-based analysis. For instance, recent studies have reported the isolation of several mycelium strains from *O*. *sinensis* fruiting bodies harvested in the wild [42,49], but identification of the fruiting bodies and mycelium species was based on morphological observations alone and no DNA analysis was provided. Given that DNA analysis of multiple barcode genes provides a reliable method for identifying fungal species, we believe that the platform established in the present study may be used, in combination with morphological observations, to identify and study *O*. *sinensis* strains as well as other fungi.

## Conclusion

Using microbiological techniques and DNA phylogenetic analysis, we have isolated and cultured the mycelium anamorph of *O*. *sinensis* fruiting bodies found in the wild. The growth conditions and chemical composition of this mycelium strain are similar to the *O*. *sinensis* fruiting bodies. Moreover, *in vitro* culture produces a mycelium that is free of contaminants (of fungal or microbial origin), pesticides, or heavy metals, and that is unadulterated—characteristics that are highly advantageous compared with some fruiting bodies available on the market. Investigations are currently under way to verify the full extent of the functional effects of the mycelium strain in laboratory animals. Further studies are also needed to verify the effects of this mycelium preparation for the prevention and treatment of human diseases.

## Supporting Information

S1 Fig Alignment of ITS-5.8S-rRNA sequences for HSM CGB999335 and *O*. *sinensis* voucher HMAS:173825. Search was performed using BLASTN. *O*. *sinensis* voucher HMAS:173825 18S ribosomal RNA gene, partial sequence; internal transcribed spacer ribosomal RNA gene, and internal transcribed spacer 2, complete sequence; and 28S ribosomal RNA gene, partial sequence. Sequence ID: EU570952.1.
(TIF)

S2 Fig Alignment of nrSSU sequences for HSM CGB999335 and *O*. *sinensis* strain SJL0809. *O*. *sinensis* strain SJL0809 18S ribosomal RNA gene, partial sequence. Sequence ID: HM135169.1.
(TIF)

S3 Fig Alignment of nrLSU sequences for HSM CGB999335 and *O*. *sinensis* strain SJL0809. *O*. *sinensis* strain SJL0809 28S ribosomal RNA gene, partial sequence. Sequence ID: HM135168.1.
(TIF)

S4 Fig Alignment of RPB1 sequences for HSM CGB999335 and *O*. *sinensis* strain EFCC 7287. *O*. *sinensis* strain EFCC 7287 DNA-dependent RNA polymerase II largest subunit (RPB1) gene, partial coding DNA sequence. Sequence ID: EF468874.1.
(TIF)

S5 Fig Alignment of RPB2 sequences for HSM CGB999335 and *O*. *sinensis* strain YN07-8. *O*. *sinensis* isolate YN07-8 DNA-dependent RNA polymerase II second largest subunit (RPB2) gene, partial. Sequence ID: JX968012.1.
(TIF)

S6 Fig Alignment of TEF-1α sequences for HSM CGB999335 and *O*. *sinensis* strain YN07-8. *O*. *sinensis* isolate YN07-8 TEF-1α gene, partial coding DNA sequence. Sequence ID: JX968017.1.
(TIF)

S7 Fig Alignment of MCM7 sequences for HSM CGB999335 and *O*. *sinensis* strain CO18. *O*. *sinensis* CO18 contig_1827, whole genome shotgun sequence. Sequence ID: ANOV01001827.1.
(TIF)

S8 Fig Alignment of β-tubulin sequences for HSM CGB999335 and *O*. *sinensis* strain CO18. *O*. *sinensis* CO18 contig_1023, whole genome shotgun sequence. Sequence ID: ANOV01001023.1.
(TIF)

S9 Fig Alignment of ATP6 sequences for HSM CGB999335 and *O*. *sinensis* strain CO18. *O*. *sinensis* CO18 contig_6466, whole genome shotgun sequence. Sequence ID: ANOV01006466.1.
(TIF)

S10 Fig Phylogenetic tree of nrSSU for *Ophiocordyceps sinensis* (*Hirsutella sinensis*) CGB999335 and related species. Evolutionary history was inferred using the neighbor-joining method and phylogenetic trees were built using the MEGA software. Bootstrap consensus tree inferred from 500 replicates is taken to represent the evolutionary history of the taxa analyzed. Branches corresponding to partitions reproduced in less than 50% bootstrap replicates are collapsed. The percentages of replicate trees in which the associated taxa clustered together in the bootstrap test (500 replicates) are shown next to the branches. Evolutionary distances were computed using the maximum composite likelihood method and are expressed as units of number of base substitutions per site.
(TIF)

S11 Fig Phylogenetic tree of nrLSU sequences for HSM CGB999335 and related species. The tree was built as in S10 Fig.
(TIF)

S12 Fig Phylogenetic tree of RPB1 sequences for HSM CGB999335 and related species. Analysis was performed as in S10 Fig.
(TIF)

S13 Fig Phylogenetic tree of RPB2 sequences for HSM CGB999335 and related species. Analysis was performed as in S10 Fig.
(TIF)

S14 Fig Phylogenetic tree of TEF-1α sequences for HSM CGB999335 and related species. Analysis was done as in S10 Fig.
(TIF)

S15 Fig Phylogenetic tree of MCM7 sequences for HSM CGB999335 and related species. Analysis was performed as in S10 Fig.
(TIF)

S16 Fig Phylogenetic tree of β-tubulin sequences for HSM CGB999335 and related species. Analysis was performed as in S10 Fig.
(TIF)

S17 Fig Phylogenetic tree of ATP6 sequences for HSM CGB999335 and related species. See S10 Fig for more information.
(TIF)

S1 Table PCR primers used in this study (DOCX)

S2 Table Specimen information and GenBank sequences used for the 5-gene phylogenetic analysis (DOCX)

S3 Table Specimen information and GenBank sequences used for the single-gene-based phylogenetic analysis (XLSX)
