# Supplementary figures and images for: Isolation, Culture and Characterization of Hirsutella sinensis Mycelium from Caterpillar Fungus Fruiting Body
Source: PLoS One. 2017 Jan 3;12(1):e0168734. doi: 10.1371/journal.pone.0168734 (PMC5207747; doi:10.1371/journal.pone.0168734)

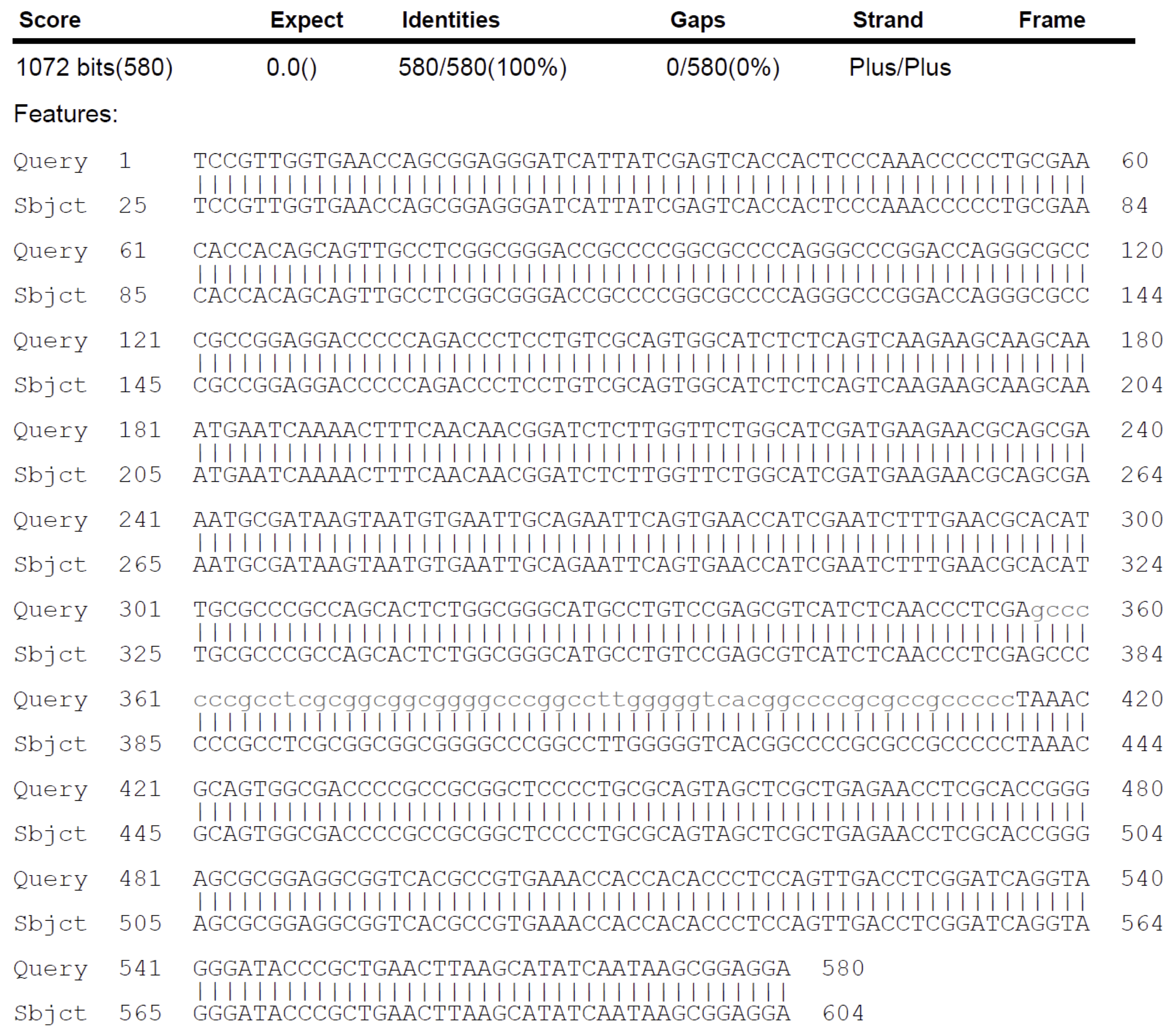

Supplement: S1 Fig — Search was performed using BLASTN. O. sinensis voucher HMAS:173825 18S ribosomal RNA gene, partial sequence; internal transcribed spacer ribosomal RNA gene, and internal transcribed spacer 2, complete sequence; and 28S ribosomal RNA gene, partial sequence. Sequence ID: EU570952.1. (TIF) [file pone.0168734.s001.tif]

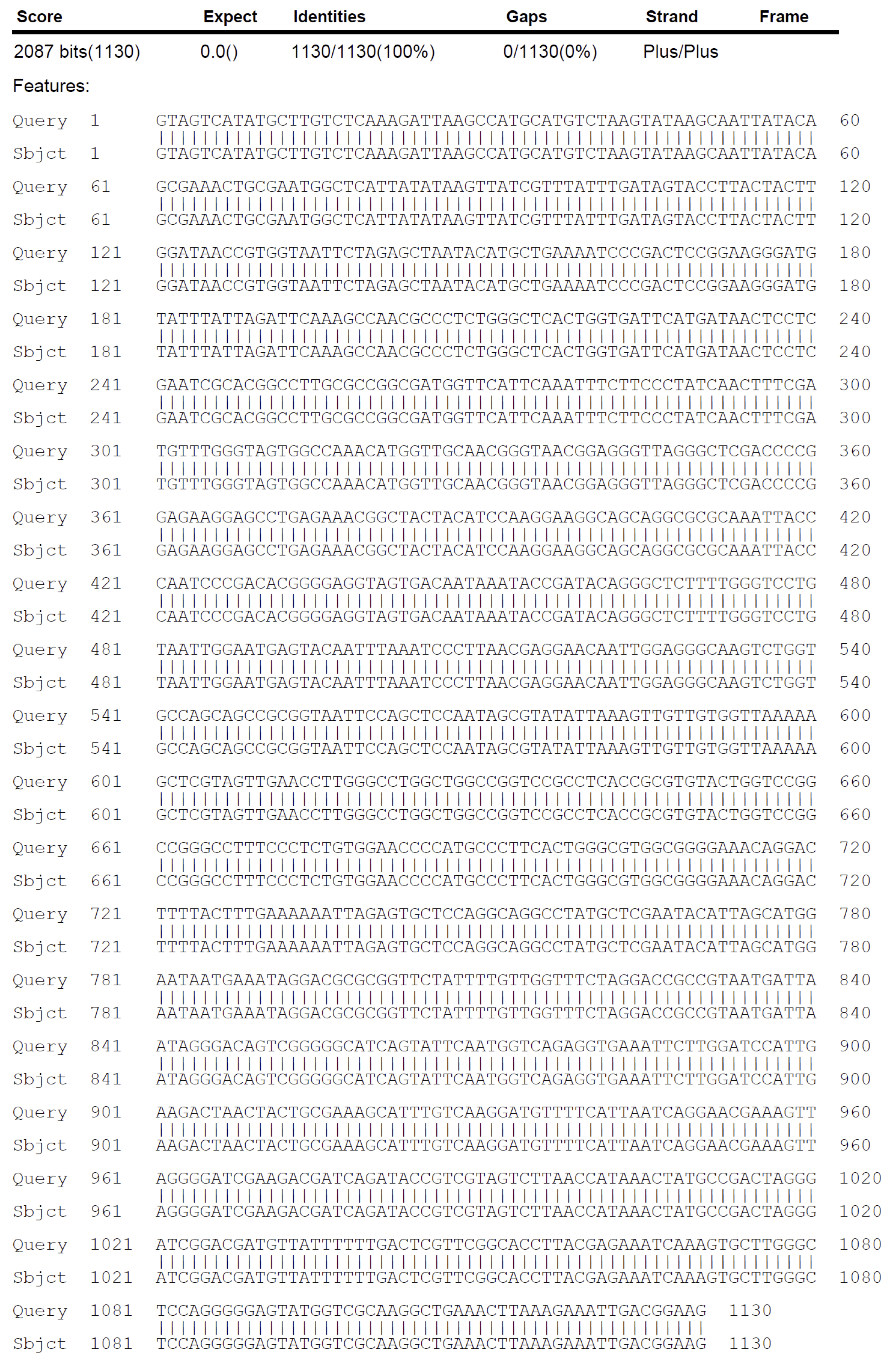

Supplement: S2 Fig — O. sinensis strain SJL0809 18S ribosomal RNA gene, partial sequence. Sequence ID: HM135169.1. (TIF) [file pone.0168734.s002.tif]

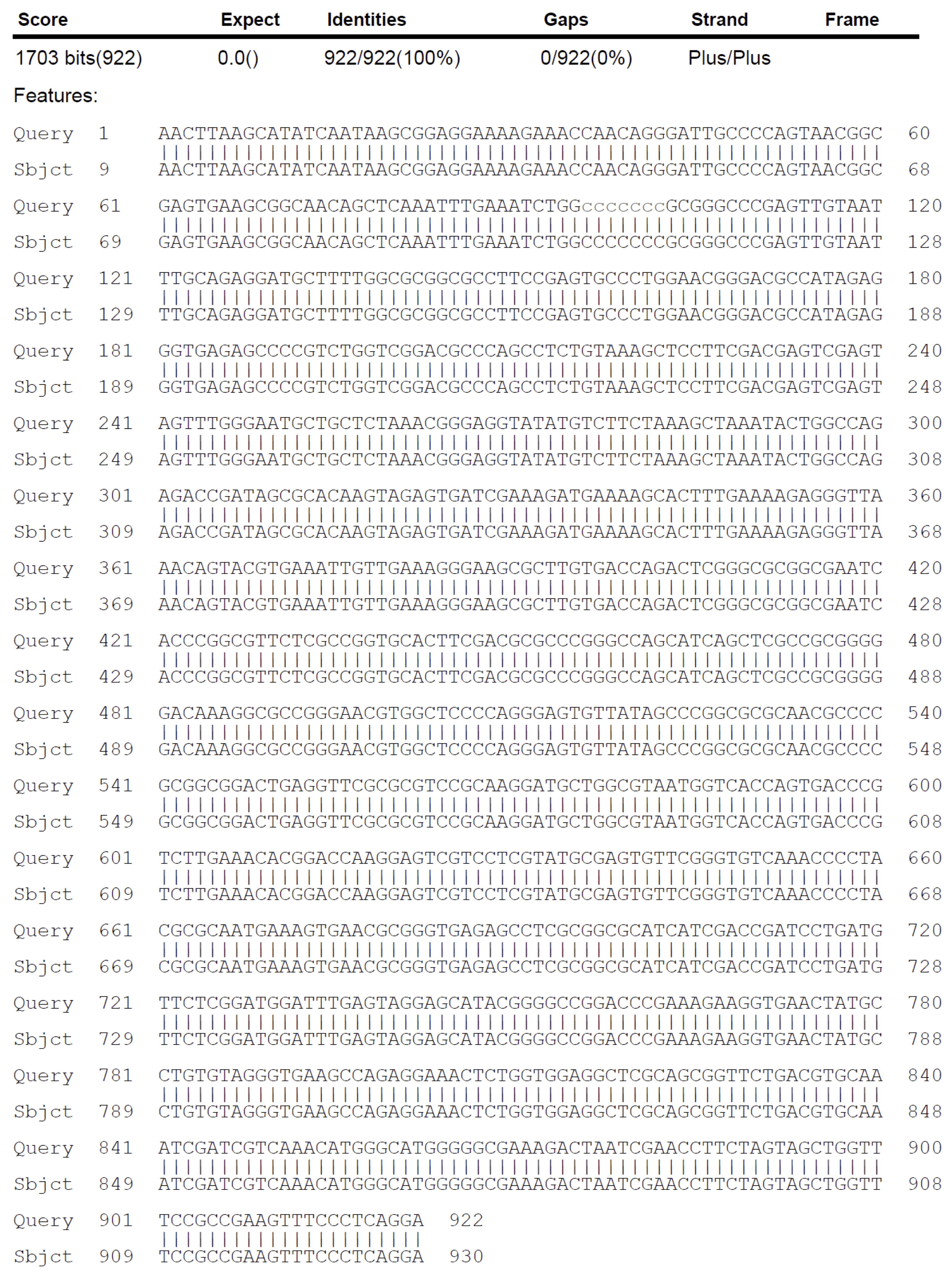

Supplement: S3 Fig — O. sinensis strain SJL0809 28S ribosomal RNA gene, partial sequence. Sequence ID: HM135168.1. (TIF) [file pone.0168734.s003.tif]

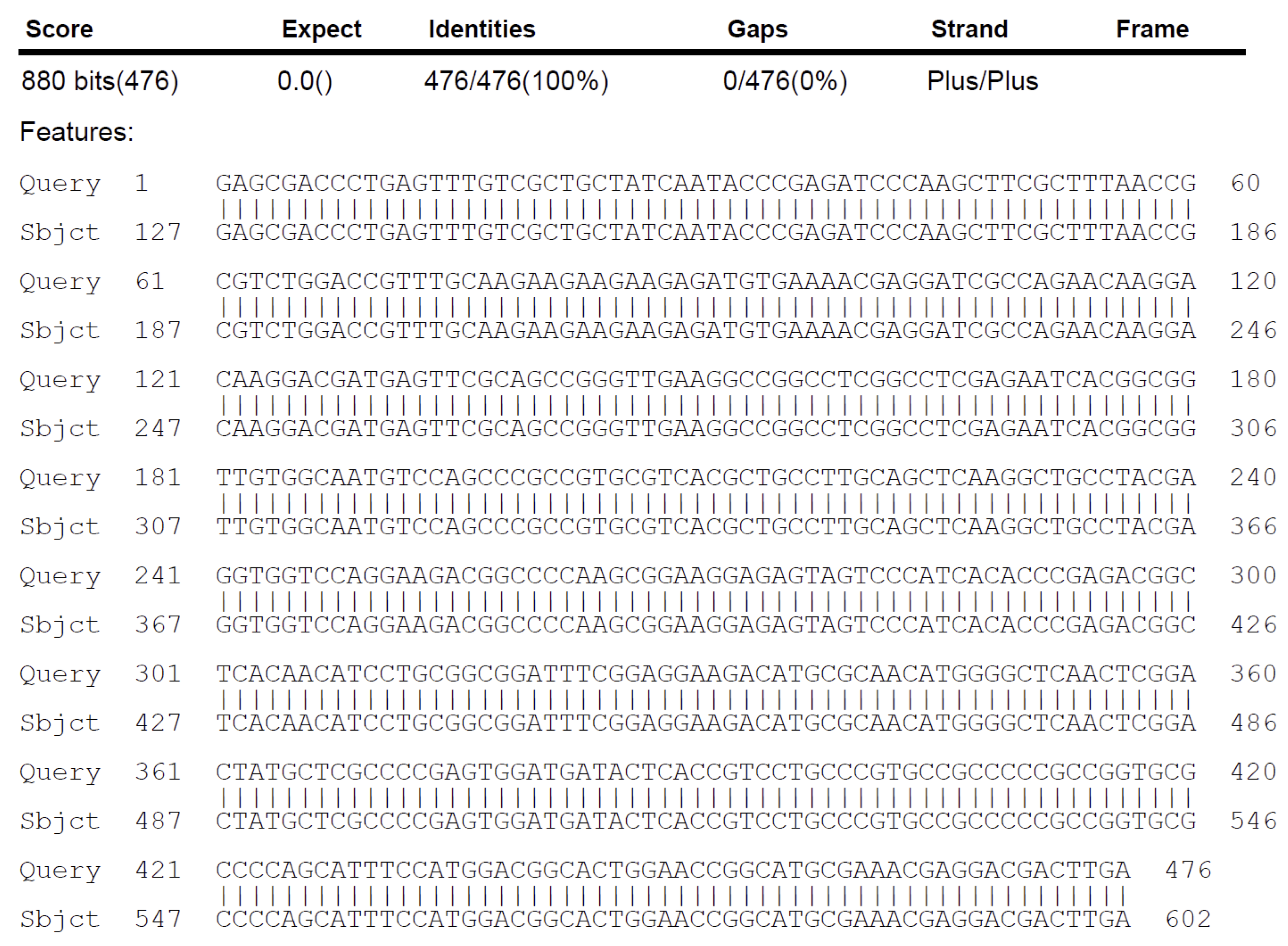

Supplement: S4 Fig — O. sinensis strain EFCC 7287 DNA-dependent RNA polymerase II largest subunit (RPB1) gene, partial coding DNA sequence. Sequence ID: EF468874.1. (TIF) [file pone.0168734.s004.tif]

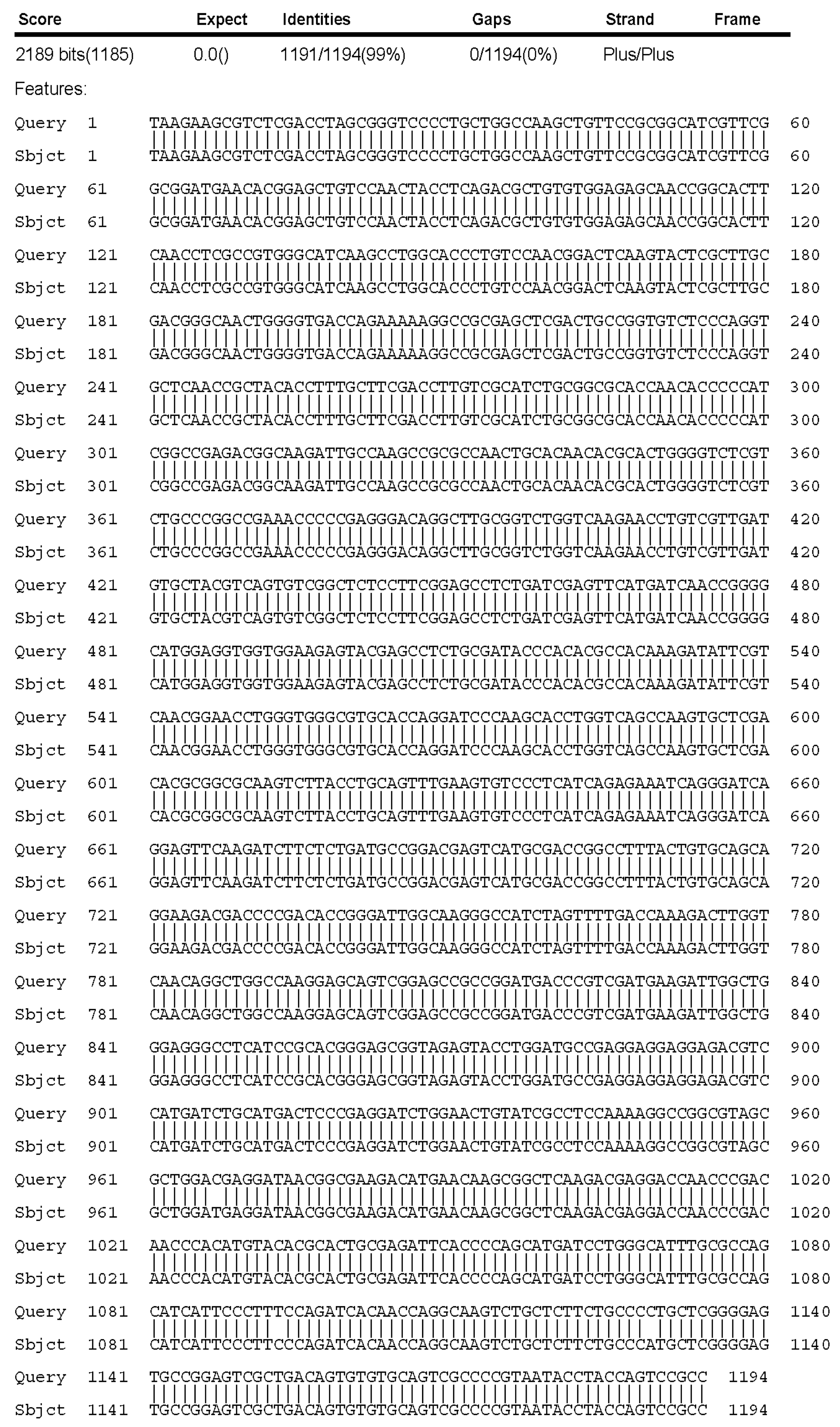

Supplement: S5 Fig — O. sinensis isolate YN07-8 DNA-dependent RNA polymerase II second largest subunit (RPB2) gene, partial. Sequence ID: JX968012.1. (TIF) [file pone.0168734.s005.tif]

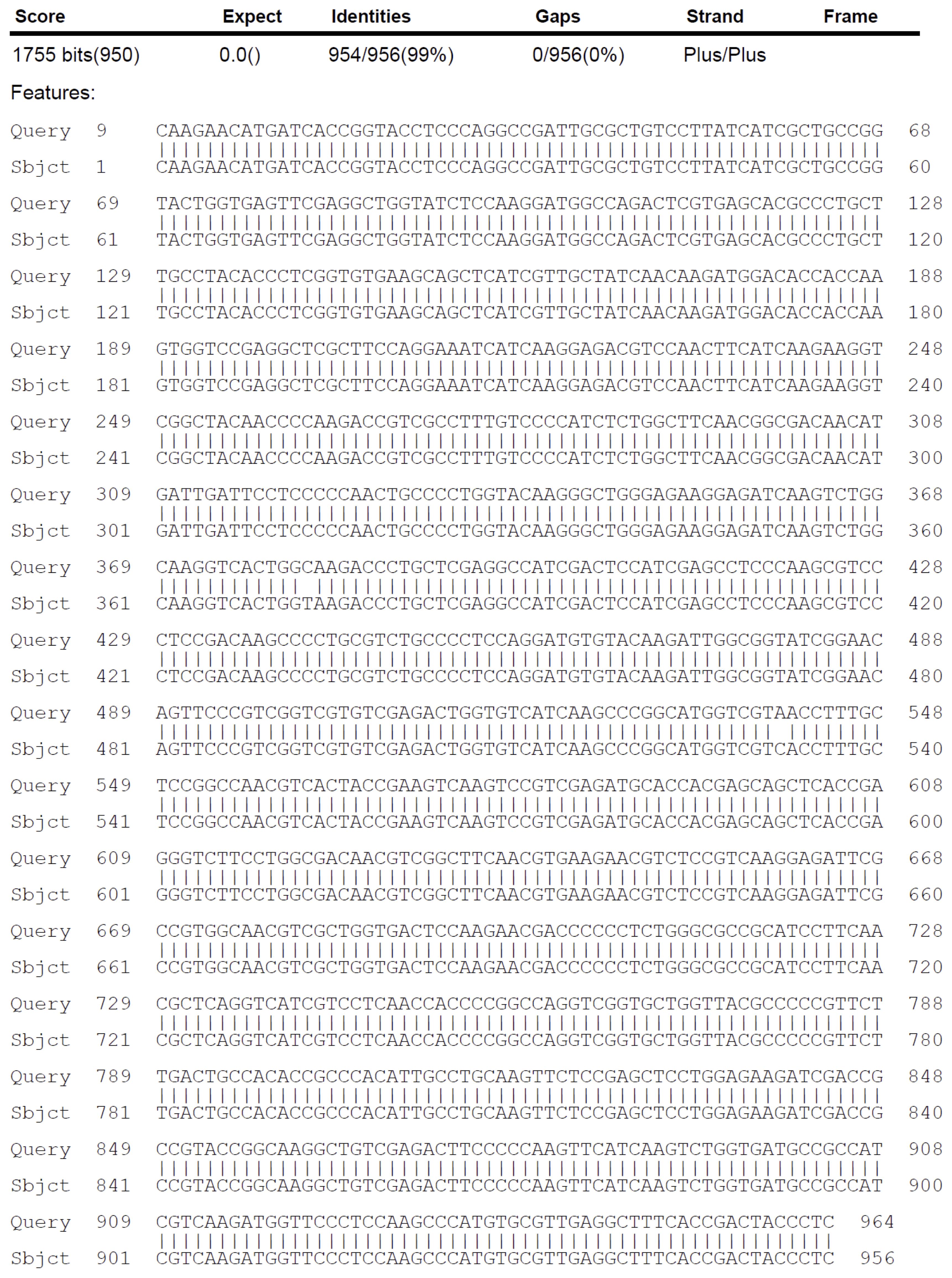

Supplement: S6 Fig — O. sinensis isolate YN07-8 TEF-1α gene, partial coding DNA sequence. Sequence ID: JX968017.1. (TIF) [file pone.0168734.s006.tif]

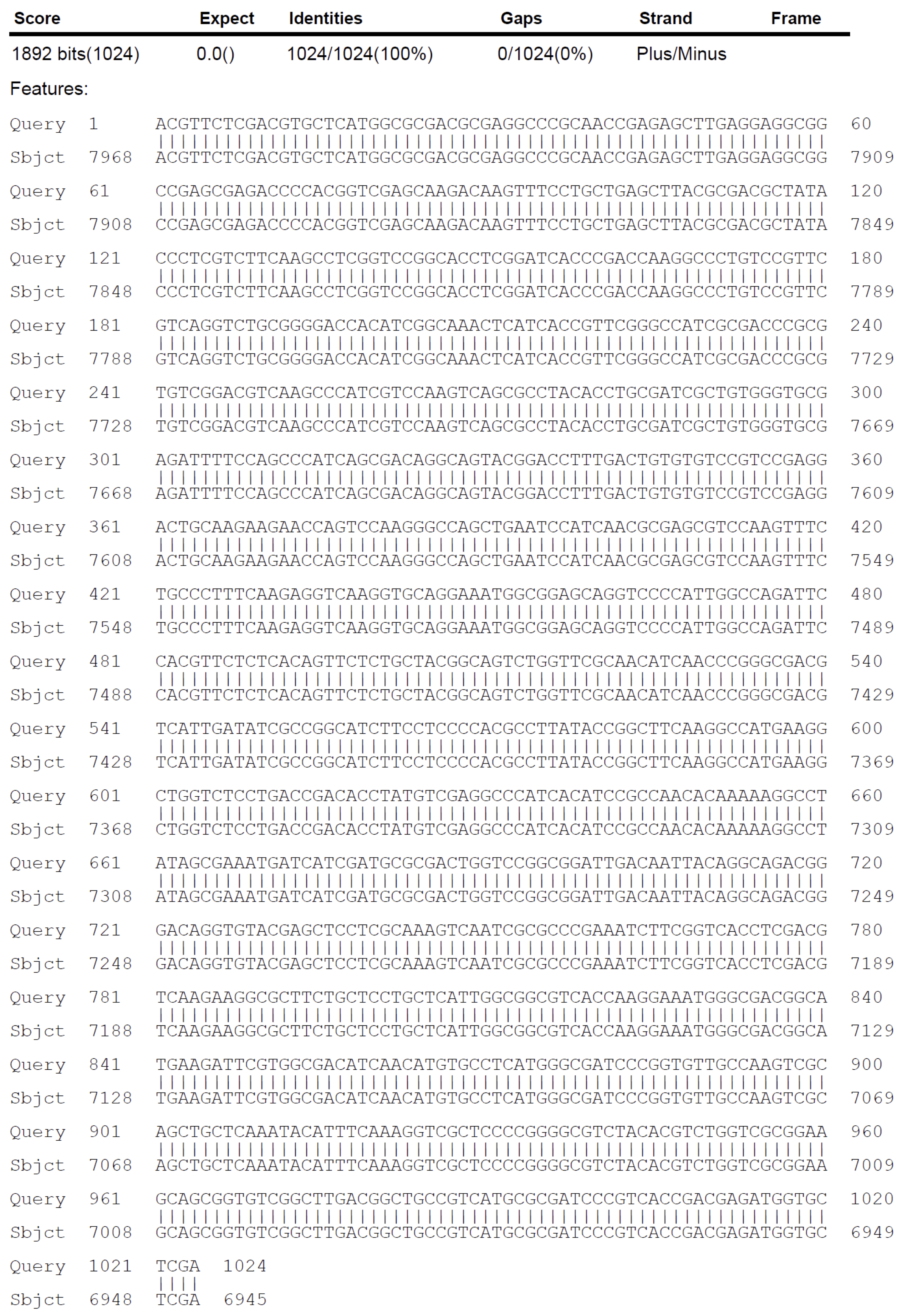

Supplement: S7 Fig — O. sinensis CO18 contig_1827, whole genome shotgun sequence. Sequence ID: ANOV01001827.1. (TIF) [file pone.0168734.s007.tif]

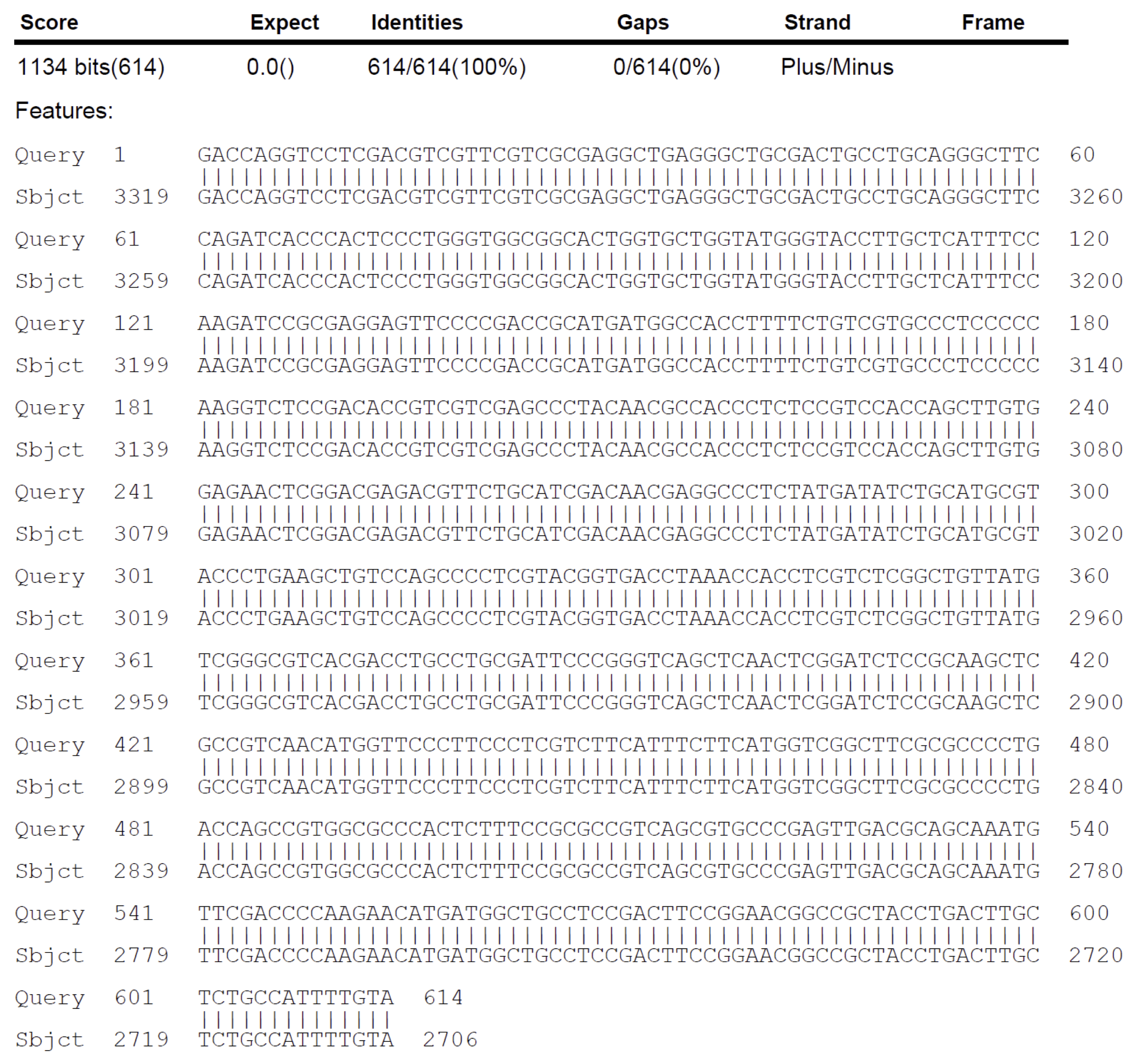

Supplement: S8 Fig — O. sinensis CO18 contig_1023, whole genome shotgun sequence. Sequence ID: ANOV01001023.1. (TIF) [file pone.0168734.s008.tif]

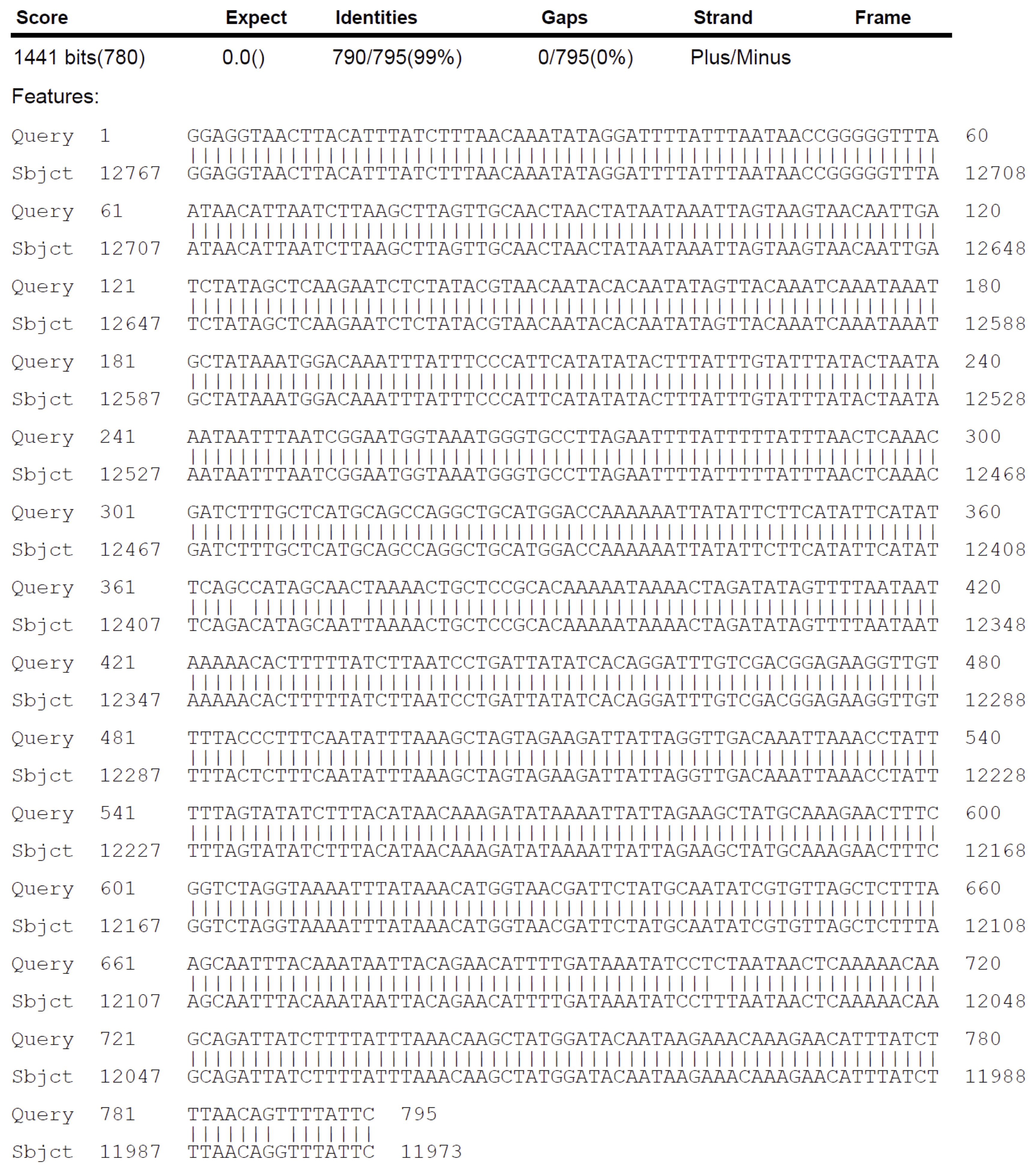

Supplement: S9 Fig — O. sinensis CO18 contig_6466, whole genome shotgun sequence. Sequence ID: ANOV01006466.1. (TIF) [file pone.0168734.s009.tif]

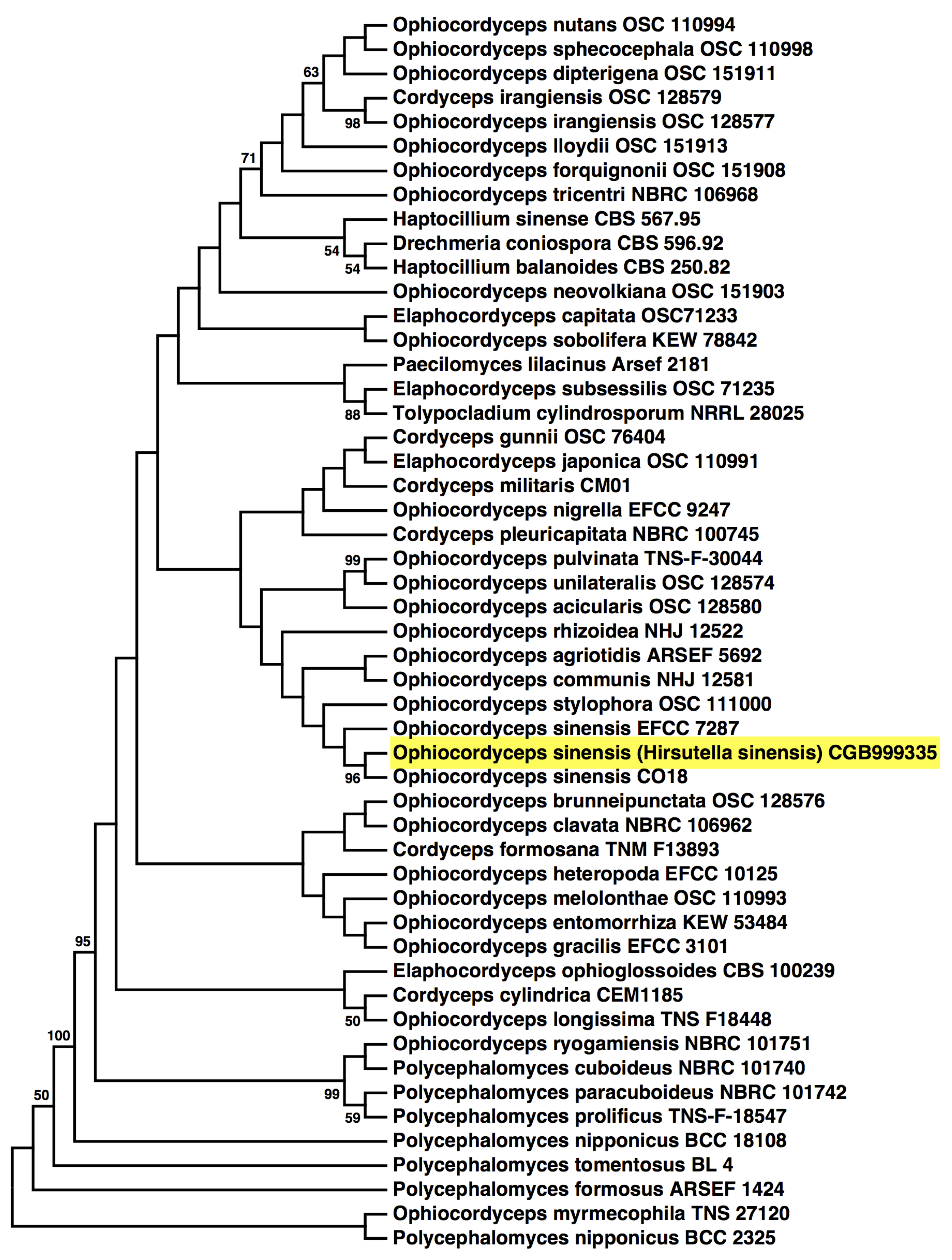

Supplement: S10 Fig — Evolutionary history was inferred using the neighbor-joining method and phylogenetic trees were built using the MEGA software. Bootstrap consensus tree inferred from 500 replicates is taken to represent the evolutionary history of the taxa analyzed. Branches corresponding to partitions reproduced in less than 50% bootstrap replicates are collapsed. The percentages of replicate trees in which the associated taxa clustered together in the bootstrap test (500 replicates) are shown next to the branches. Evolutionary distances were computed using the maximum composite likelihood method and are expressed as units of number of base substitutions per site. (TIF) [file pone.0168734.s010.tif]

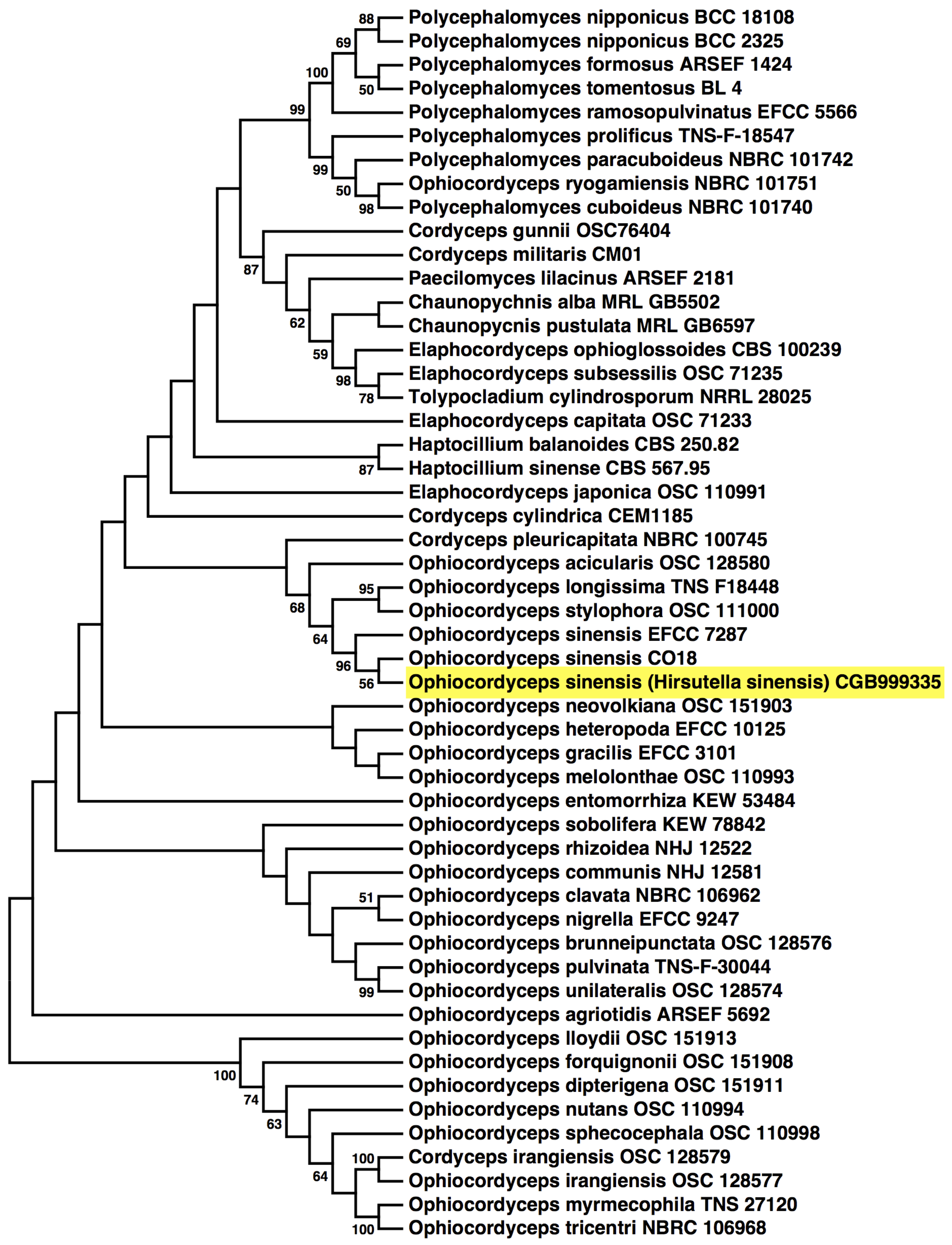

Supplement: S11 Fig — The tree was built as in S10 Fig. (TIF) [file pone.0168734.s011.tif]

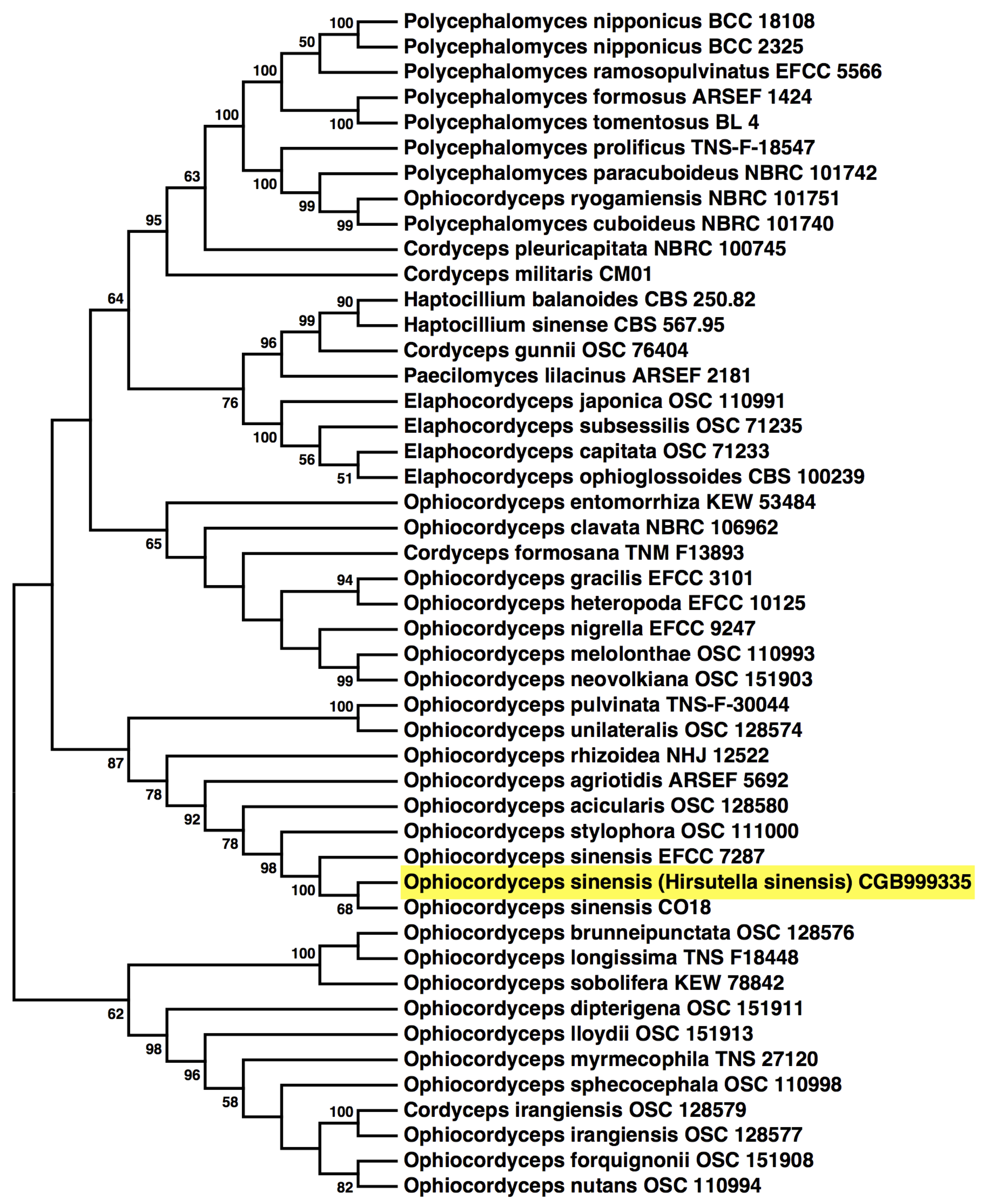

Supplement: S12 Fig — Analysis was performed as in S10 Fig. (TIF) [file pone.0168734.s012.tif]

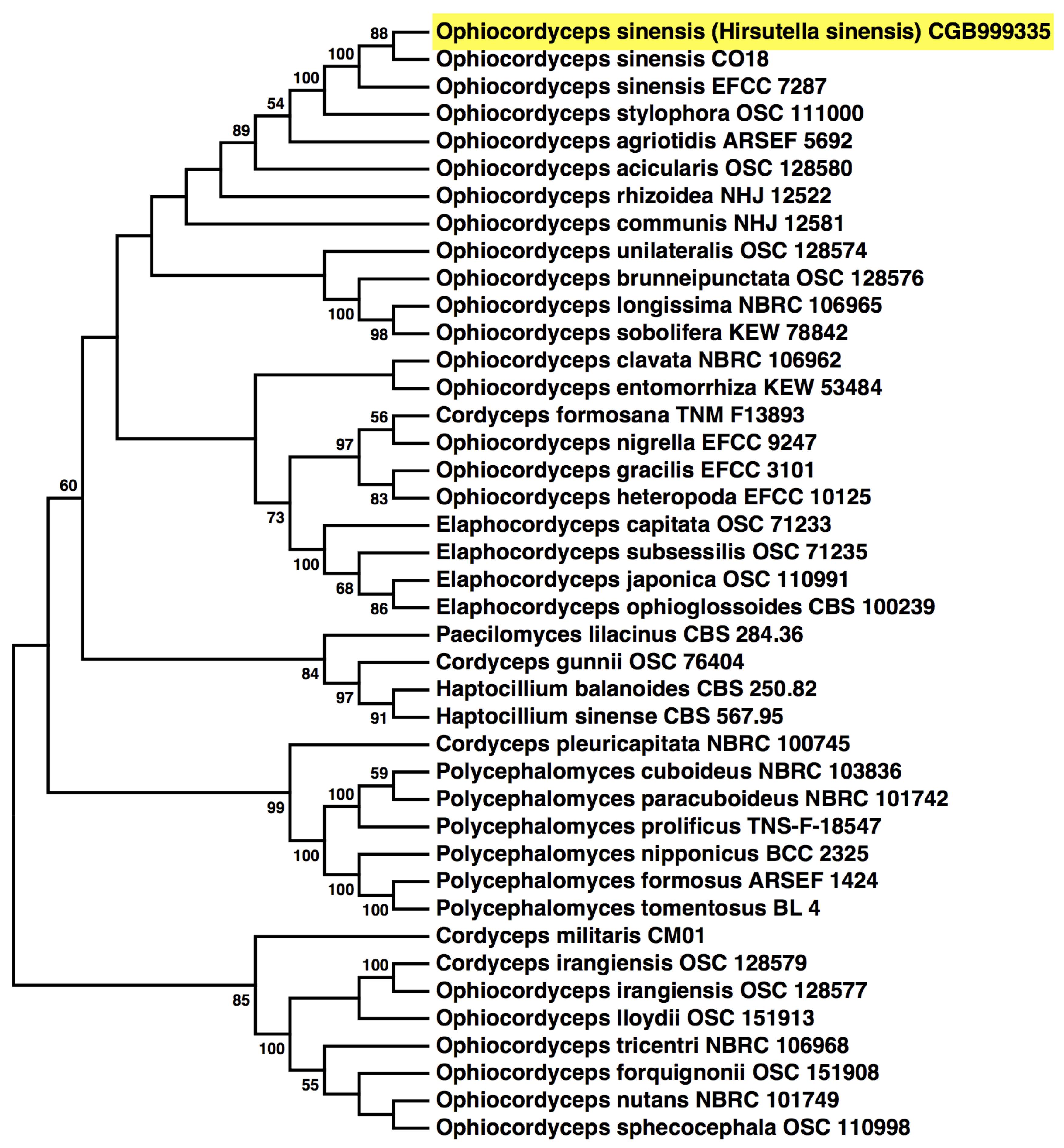

Supplement: S13 Fig — Analysis was performed as in S10 Fig. (TIF) [file pone.0168734.s013.tif]

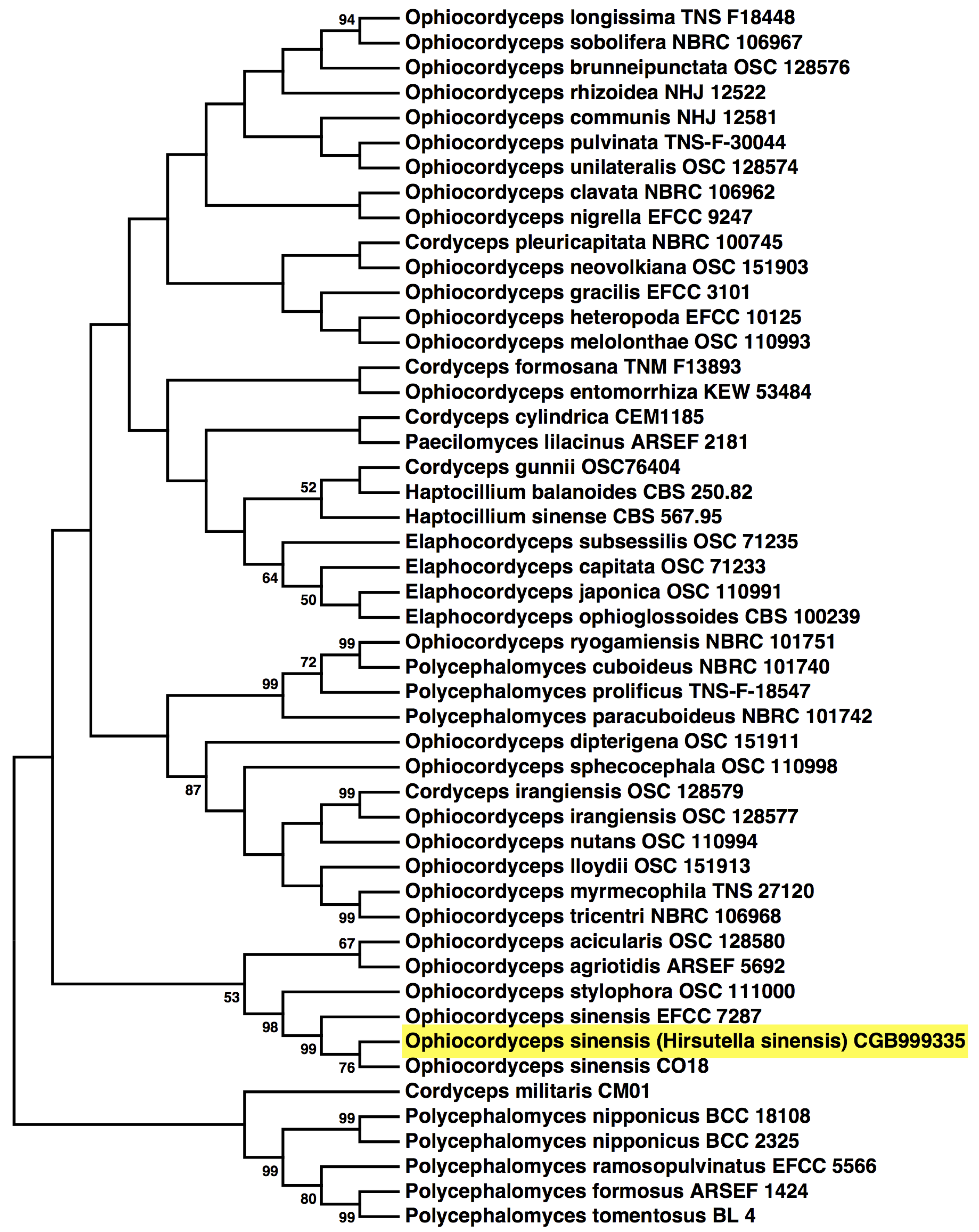

Supplement: S14 Fig — Analysis was done as in S10 Fig. (TIF) [file pone.0168734.s014.tif]

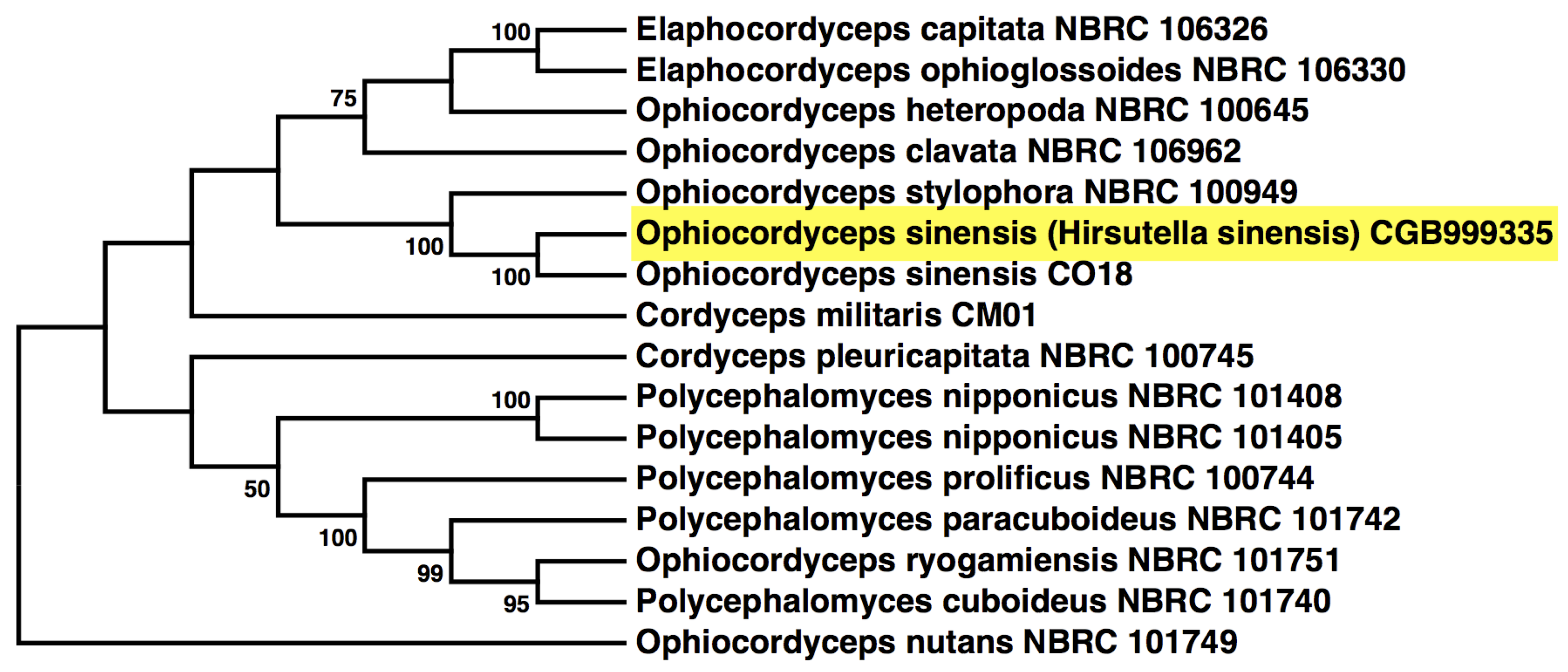

Supplement: S15 Fig — Analysis was performed as in S10 Fig. (TIF) [file pone.0168734.s015.tif]

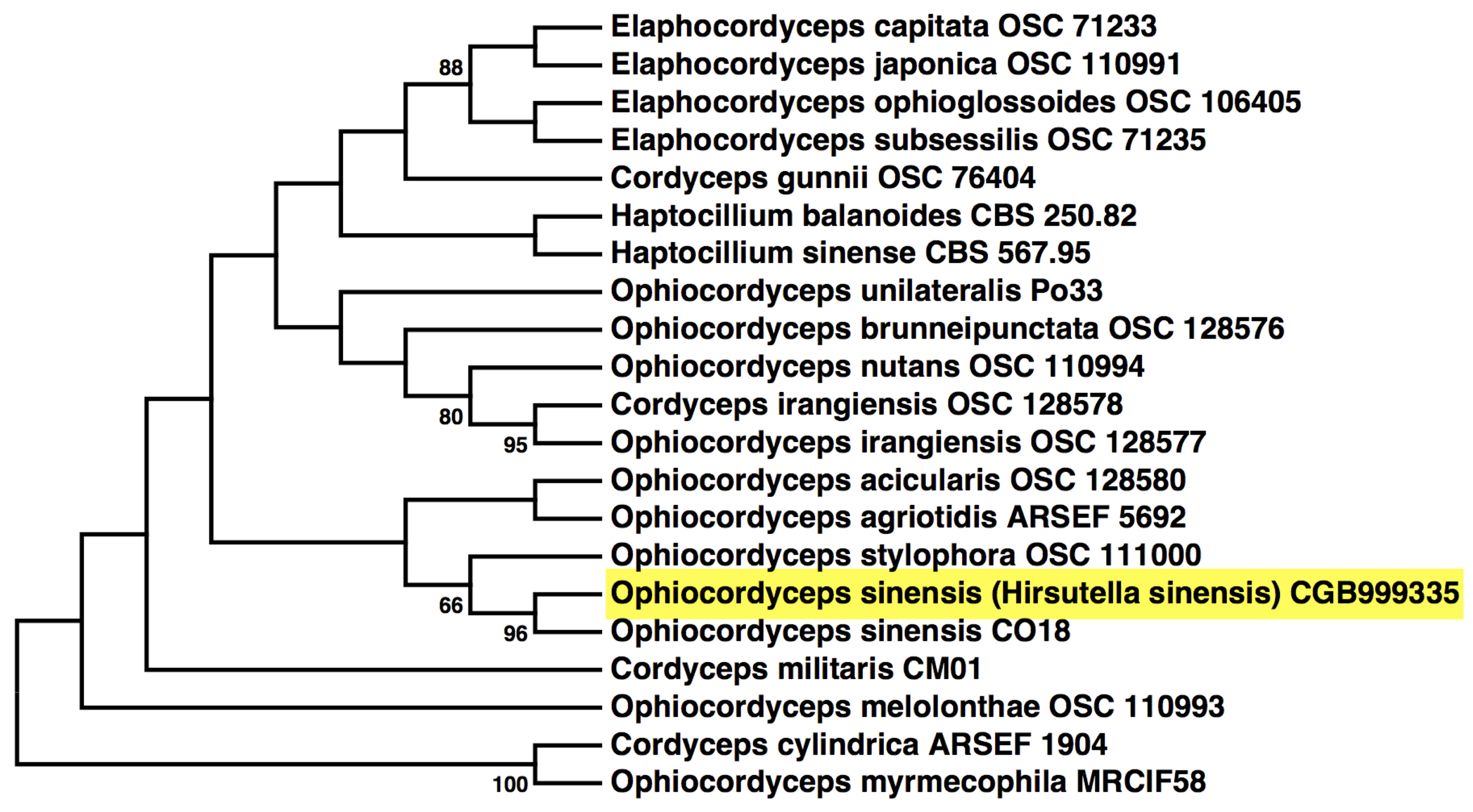

Supplement: S16 Fig — Analysis was performed as in S10 Fig. (TIF) [file pone.0168734.s016.tif]

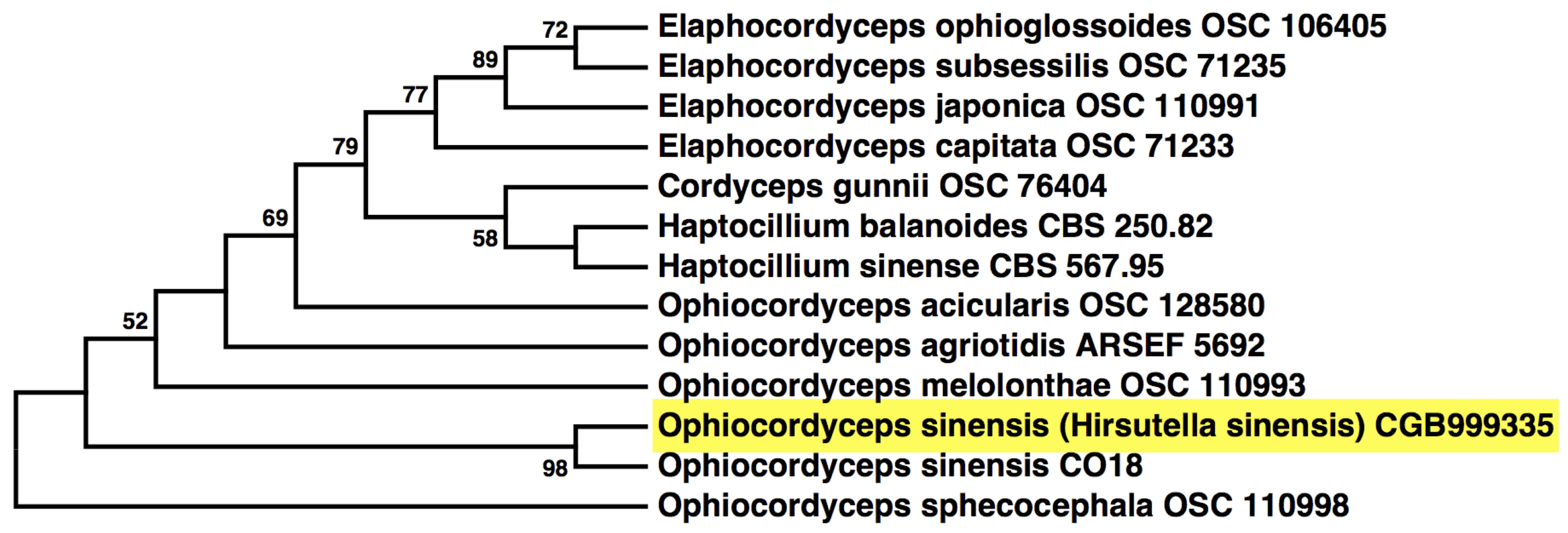

Supplement: S17 Fig — See S10 Fig for more information. (TIF) [file pone.0168734.s017.tif]
