# Supplementary material for: Isolation, Culture and Characterization of Hirsutella sinensis Mycelium from Caterpillar Fungus Fruiting Body
Source: PLoS One. 2017 Jan 3;12(1):e0168734. doi: 10.1371/journal.pone.0168734 (PMC5207747; doi:10.1371/journal.pone.0168734)
Supplement: S1 Table — (DOCX) [file pone.0168734.s018.docx]

| **S1 Table. PCR primers used in this study.** | | | | |
| --- | --- | --- | --- | --- |
| **Gene** | **Primer** | **Sequence (5'-3')** | **Direction** | **Reference** |
| *ITS* | ITS5 | GGAAGTAAAAGTCGTAACAAGG | Forward | White et al. (1990) [[1](#_ENREF_1)] |
|  | ITS4 | TCCTCCGCTTATTGATATGC | Reverse |  |
| *nrSSU* | NS1 | GTAGTCATATGCTTGTCTC | Forward | White et al. (1990) [[1](#_ENREF_1)] |
|  | NS4 | CTTCCGTCAATTCCTTTAAG | Reverse |  |
| *nrLSU* | LR0R | GTACCCGCTGAACTTAAGC | Forward | Vilgalys and Sun (1994) [[2](#_ENREF_2)] |
|  | LR5 | ATCCTGAGGGAAACTTC | Reverse |  |
| *RPB1* | RPB1Af | GARTGYCCDGGDCAYTTYGG | Forward | Castlebury et al. (2004) [[3](#_ENREF_3)] |
|  | RPB1Cr | CCNGCDATNTCRTTRTCCATRTA | Reverse |  |
| *RPB2* | fRPB2-5F | GAYGAYMGWGATCAYTTYGG | Forward | Liu et al. (1999) [[4](#_ENREF_4)] |
|  | fRPB2-7cR | CCCATRGCTTGTYYRCCCAT | Reverse |  |
| *MCM7* | 709F | ACIMGIGTITCVGAYGTHAARCC | Forward | Schoch et al. (2012) [[5](#_ENREF_5)] |
|  | 1348R | GAYTTDGCIACICCIGGRTCWCCCAT | Reverse |  |
| *β-tubulin* | T12 | TAACAACTGCTGGGCCAAGGGTCAC | Forward | O’Donnell and Cigelnik (1997) [[6](#_ENREF_6)] |
|  | T22 | TCTGGATGTTGTTGGGAATCC | Reverse |  |
| *EF-1α* | 983F | GCYCCYGGHCAYCGTGAYTTYAT | Forward | Sung et al. (2007) [[7](#_ENREF_7)] |
|  | 2218R | ATGACACCRACRGCRACRGTYTG | Reverse |  |
| *mtATP6* | ATPC1A | AGAWCAATTYGAARTRAGAG | Forward | Castlebury et al. (2004) [[3](#_ENREF_3)] |
|  | ATPC2A | ACAAAYACTTGWGCTTGKATWAAIGC | Reverse |  |

**References**

1. White TJ, Bruns TD, Lee S, Taylor J (1990) Amplification and direct sequencing of fungal ribosomal rna genes for phylogenetics. In: Innis MA, Gelfand DH, Sninsky JJ, White TJ, editors. Pcr protocols, a guide to methods and applications. San Diego: Academic Press. pp. 315–322.

2. Vilgalys R, Sun BL (1994) Ancient and recent patterns of geographic speciation in the oyster mushroom pleurotus revealed by phylogenetic analysis of ribosomal DNA sequences. Proc Natl Acad Sci U S A 91: 4599–4603.

3. Castlebury LA, Rossman AY, Sung GH, Hyten AS, Spatafora JW (2004) Multigene phylogeny reveals new lineage for stachybotrys chartarum, the indoor air fungus. Mycol Res 108: 864–872.

4. Liu YJ, Whelen S, Hall BD (1999) Phylogenetic relationships among ascomycetes: Evidence from an rna polymerse ii subunit. Mol Biol Evol 16: 1799–1808.

5. Schoch CL, Seifert KA, Huhndorf S, Robert V, Spouge JL, et al. (2012) Nuclear ribosomal internal transcribed spacer (its) region as a universal DNA barcode marker for fungi. Proc Natl Acad Sci U S A 109: 6241–6246.

6. O'Donnell K, Cigelnik E (1997) Two divergent intragenomic rdna its2 types within a monophyletic lineage of the fungus fusarium are nonorthologous. Mol Phylogenet Evol 7: 103–116.

7. Sung GH, Sung JM, Hywel-Jones NL, Spatafora JW (2007) A multi-gene phylogeny of clavicipitaceae (ascomycota, fungi): Identification of localized incongruence using a combinational bootstrap approach. Mol Phylogenet Evol 44: 1204–1223.
