# Supplementary material for: Isolation, Culture and Characterization of Hirsutella sinensis Mycelium from Caterpillar Fungus Fruiting Body
Source: PLoS One. 2017 Jan 3;12(1):e0168734. doi: 10.1371/journal.pone.0168734 (PMC5207747; doi:10.1371/journal.pone.0168734)
Supplement: S2 Table — (DOCX) [file pone.0168734.s019.docx]

| **S2 Table. Specimen information and GenBank sequences used for the 5-gene phylogenetic analysis** | | | | | | | |
| --- | --- | --- | --- | --- | --- | --- | --- |
| **Family** | **Species** | **Voucher Information** | **nrSSU** | **nrLSU** | **RPB1** | **RPB2** | **EF-1α** |
| Bionectriaceae | *Bionectria ochroleuca* | CBS 114056 |  |  |  | DQ522415 |  |
|  | *Hydropisphaera peziza* | GJS 92-101 | AY489698 | AY489730 | AY489661 |  | AY489625 |
|  | *Hydropisphaera peziza* | CBS 102038 |  |  |  | DQ522444 |  |
|  | *Hydropisphaera erubescens* | ATCC 36093 | AY545722 | AY545726 | DQ522390 | AY545731 | DQ522344 |
|  | *Roumegueriella rufula* | CBS 346.85 | DQ522561 | DQ518776 | DQ522403 | DQ522461 | DQ522355 |
|  | *Roumegueriella rufula* | GJS 91-164 | EF469129 | EF469082 | EF469099 | EF469116 | EF469070 |
| Hypocreaceae | *Aphysiostroma stercorarium* | ATCC 62321 | AF543769 | AF543792 | AY489633 | EF469103 | AF543782 |
|  | *Hypocrea lutea* | ATCC 208838 | AF543768 | AF543791 | AY489662 | DQ522446 | AF543781 |
|  | *Hypomyces polyporinus* | ATCC 76479 | AF543771 | AF543793 | AY489663 |  | AF543784 |
|  | *Sphaerostilbella berkeleyana* | CBS 102308 |  |  | AY489671 | DQ522465 |  |
|  | *Verticillium incurvum* | CBS 460.88 | AF339600 | AF339551 | DQ522410 | DQ522470 | DQ522362 |
| Nectriaceae | *Cosmospora coccinea* | CBS 114050 |  | GQ505990 | GQ506020 | DQ522438 |  |
|  | *Gliocephalotrichum bulbilium* | ATCC 22228 | AY489700 | AY489732 | AY489664 | EF469114 |  |
|  | *Nectria cinnabarina* | CBS 114055 |  |  |  | DQ522456 | AY489627 |
|  | *Ophionectria trichospora* | CBS 109876 | AF543766 | AF543790 | AY489669 | DQ522457 | AF543779 |
|  | *Pseudonectria rousseliana* | CBS 114049 |  | JF937575 |  | DQ522459 |  |
|  | *Viridispora diparietispora* | CBS 102797 |  |  |  | DQ522471 |  |
| Ophiocordycipitaceae | *Chaunopycnis alba* | MRL GB5502 |  | AF245297 |  |  |  |
|  | *Chaunopycnis alba* | MRL MF6799 |  | AF373284 |  |  |  |
|  | *Chaunopycnis pustulata* | MRL GB6597 |  | AF389190 |  |  |  |
|  | *Chaunopycnis pustulata* | MRL MF5368LR |  | AF373282 |  |  |  |
|  | *Cordyceps cf. acicularis* | OSC 128580 | DQ522543 | DQ518757 | DQ522371 | DQ522423 | DQ522326 |
|  | *Cordyceps cylindrica* | CEM 1185 | KJ878907 | KJ878872 |  |  | KJ878955 |
|  | *Cordyceps formosana* | TNM F13893 | KJ878908 |  | KJ878988 | KJ878943 | KJ878956 |
|  | *Cordyceps gunnii* | OSC 76404 | AF339572 | AF339522 | AY489650 | DQ522426 | AY489616 |
|  | *Cordyceps irangiensis* | OSC 128579 | EF469123 | EF469076 | EF469089 | EF469107 | EF469060 |
|  | *Cordyceps militaris* | NBRC 100741 | JN941755 | JN941386 | JN992489 | AB968567 | AB968606 |
|  | *Cordyceps militaris* | OSC 93623 | AY184977 | AY184966 | DQ522377 |  | DQ522332 |
|  | *Cordyceps pleuricapitata* | NBRC 100746 | KF049607 | KF049625 | KF049643 | KF049668 | KF049680 |
|  | *Cordyceps pleuricapitata* | NBRC 100745 | KF049606 | KF049624 | KF049642 | KF049667 | KF049679 |
|  | *Drechmeria coniospora* | CBS 596.92 | AF106012 |  |  |  |  |
|  | *Elaphocordyceps capitata* | OSC 71233 | AY489689 | AY489721 | AY489649 | DQ522421 | AY489615 |
|  | *Elaphocordyceps fracta* | OSC 110990 | DQ522545 | DQ518759 | DQ522373 | DQ522425 | DQ522328 |
|  | *Elaphocordyceps japonica* | OSC 110991 | DQ522547 | DQ518761 | DQ522375 | DQ522428 | DQ522330 |
|  | *Elaphocordyceps longisegmentis* | OSC 110992 |  | EF468816 | EF468864 | EF468919 |  |
|  | *Elaphocordyceps ophioglossoides* | OSC 106405 | AY489691 | AY489723 | AY489652 | DQ522429 | AY489618 |
|  | *Elaphocordyceps ophioglossoides* | CBS 100239 | KJ878910 | KJ878874 | KJ878990 | KJ878944 | KJ878958 |
|  | *Elaphocordyceps subsessilis* | OSC 71235 | EF469124 | EF469077 | EF469090 | EF469108 | EF469061 |
|  | *Haptocillium balanoides* | CBS 250.82 | AF339588 | AF339539 | DQ522388 | DQ522442 | DQ522342 |
|  | *Haptocillium sinense* | CBS 567.95 | AF339594 | AF339545 | DQ522389 | DQ522443 | DQ522343 |
|  | *Haptocillium zeosporum* | CBS 335.8 | AF339589 | AF339540 | EF469091 | EF469109 | EF469062 |
|  | *Harposporium anguillulae* | ARSEF 5593 |  | AY636081 |  |  |  |
|  | *Harposporium anguillulae* | ARSEF 5407 |  | AY636080 |  |  |  |
|  | *Harposporium helicoides* | ARSEF 5354 | AF339577 | AF339527 |  |  |  |
|  | *Hirsutella crinalis* | TNS F18550 | KJ878911 | KJ878875 |  |  | KJ878959 |
|  | *Hirsutella sp.* | NHJ 12525 | EF469125 | EF469078 | EF469092 | EF469111 | EF469063 |
|  | *Hirsutella sp.* | OSC 128575 | EF469126 | EF469079 | EF469093 | EF469110 | EF469064 |
|  | *Hymenostilbe aurantiaca* | OSC 128578 | DQ522556 | DQ518770 | DQ522391 | DQ522445 | DQ522345 |
|  | *Hymenostilbe muscaria* | OSC 151902 | KJ878912 | KJ878876 | KJ878991 | KJ878945 |  |
|  | *Hymenostilbe odonatae* | TNS F27117 |  | KJ878878 |  |  |  |
|  | *Hymenostilbe odonatae* | TNS F18563 |  | KJ878877 | KJ878992 |  |  |
|  | *Isaria takamizuensis* | NHJ 3582 | EU369097 | EU369034 |  |  | EU369015 |
|  | *Isariata kamizusanensis* | NHJ 3497 | EU369096 | EU369033 | EU369053 | EU369074 | EU369014 |
|  | *Nomuraea atypicola* | RCEF 3833 | KJ878913 | KJ878879 | KJ878993 |  | KJ878960 |
|  | *Nomuraea atypicola* | OSC 151901 | KJ878914 | KJ878880 | KJ878994 |  | KJ878961 |
|  | *Nomuraea atypicola* | CBS 744.73 | EF468987 | EF468841 | EF468892 |  | EF468786 |
|  | *Ophiocordyceps acicularis* | OSC 110987 | EF468950 | EF468805 | EF468852 |  | EF468744 |
|  | *Ophiocordyceps acicularis* | OSC 110988 | EF468951 | EF468804 | EF468853 |  | EF468745 |
|  | *Ophiocordyceps acicularis* | OSC 128580 | DQ522543 | DQ518757 | DQ522371 | DQ522423 | DQ522326 |
|  | *Ophiocordyceps agriotidis* | ARSEF 5692 | DQ522540 | DQ518754 | DQ522368 | DQ522418 | DQ522322 |
|  | *Ophiocordyceps annulata* | CEM 303 | KJ878915 | KJ878881 | KJ878995 |  | KJ878962 |
|  | *Ophiocordyceps aphodii* | ARSEF 5498 | DQ522541 | DQ518755 |  | DQ522419 | DQ522323 |
|  | *Ophiocordyceps brunneipunctata* | OSC 128576 | DQ522542 | DQ518756 | DQ522369 | DQ522420 | DQ522324 |
|  | *Ophiocordyceps clavata* | CEM 1763 |  | KJ878883 | KJ878997 |  | KJ878964 |
|  | *Ophiocordyceps clavata* | NBRC 106961 | JN941727 | JN941414 | JN992461 |  |  |
|  | *Ophiocordyceps clavata* | NBRC 106962 | JN941726 | JN941415 | JN992460 |  |  |
|  | *Ophiocordyceps clavata* | CEM 1762 | KJ878916 | KJ878882 | KJ878996 |  | KJ878963 |
|  | *Ophiocordyceps communis* | NHJ 12582 | EF468975 | EF468830 |  | EF468926 | EF468771 |
|  | *Ophiocordyceps communis* | NHJ 12581 | EF468973 | EF468831 |  | EF468930 | EF468775 |
|  | *Ophiocordyceps curculionum* | OSC 151910 | KJ878918 | KJ878885 | KJ878999 |  |  |
|  | *Ophiocordyceps dipterigena* | OSC 151912 | KJ878920 | KJ878887 | KJ879001 |  | KJ878967 |
|  | *Ophiocordyceps dipterigena* | OSC 151911 | KJ878919 | KJ878886 | KJ879000 |  | KJ878966 |
|  | *Ophiocordyceps elongata* | OSC 110989 |  | EF468808 | EF468856 |  | EF468748 |
|  | *Ophiocordyceps entomorrhiza* | KEW 53484 | EF468954 | EF468809 | EF468857 | EF468911 | EF468749 |
|  | *Ophiocordyceps formicarum* | TNS F18565 | KJ878921 | KJ878888 | KJ879002 | KJ878946 | KJ878968 |
|  | *Ophiocordyceps forquignonii* | OSC 151908 | KJ878922 | KJ878889 | KJ879003 | KJ878947 |  |
|  | *Ophiocordyceps gracilis* | EFCC 3101 | EF468955 | EF468810 | EF468858 | EF468913 | EF468750 |
|  | *Ophiocordyceps gracilis* | OSC 151906 | KJ878923 | KJ878890 |  |  | KJ878969 |
|  | *Ophiocordyceps gracilis* | EFCC 8572 | EF468956 | EF468811 | EF468859 | EF468912 | EF468751 |
|  | *Ophiocordyceps heteropoda* | EFCC 10125 | EF468957 | EF468812 | EF468860 | EF468914 | EF468752 |
|  | *Ophiocordyceps irangiensis* | OSC 128577 | DQ522546 | DQ518760 | DQ522374 | DQ522427 | DQ522329 |
|  | *Ophiocordyceps konnoana* | EFCC 7295 | EF468958 |  | EF468862 | EF468915 |  |
|  | *Ophiocordyceps konnoana* | EFCC 7315 | EF468959 |  | EF468861 | EF468916 | EF468753 |
|  | *Ophiocordyceps lloydii* | OSC 151913 | KJ878924 | KJ878891 | KJ879004 | KJ878948 | KJ878970 |
|  | *Ophiocordyceps longissima* | EFCC 6814 |  | EF468817 | EF468865 |  | EF468757 |
|  | *Ophiocordyceps longissima* | TNS F18448 | KJ878925 | KJ878892 | KJ879005 |  | KJ878971 |
|  | *Ophiocordyceps melolonthae* | OSC 110993 | DQ522548 | DQ518762 | DQ522376 |  | DQ522331 |
|  | *Ophiocordyceps myrmecophila* | HMAS_199620 | KJ878927 | KJ878893 | KJ879007 |  | KJ878973 |
|  | *Ophiocordyceps myrmecophila* | TNS 27120 | KJ878929 | KJ878895 | KJ879009 |  | KJ878975 |
|  | *Ophiocordyceps myrmecophila* | CEM 1710 | KJ878928 | KJ878894 | KJ879008 |  | KJ878972 |
|  | *Ophiocordyceps neovolkiana* | OSC 151903 | KJ878930 | KJ878896 | KJ879010 |  | KJ878976 |
|  | *Ophiocordyceps nigrella* | EFCC 9247 | EF468963 | EF468818 | EF468866 | EF468920 | EF468758 |
|  | *Ophiocordyceps nutans* | OSC 110994 | DQ522549 | DQ518763 | DQ522378 |  | DQ522333 |
|  | *Ophiocordyceps pruinosa* | NHJ 12994 | EU369106 | EU369041 | EU369063 | EU369084 | EU369024 |
|  | *Ophiocordyceps purpureostromata* | TNS F18430 | KJ878931 | KJ878897 | KJ879011 |  | KJ878977 |
|  | *Ophiocordyceps ravenelii* | OSC 151914 | KJ878932 |  | KJ879012 | KJ878950 | KJ878978 |
|  | *Ophiocordyceps ravenelii* | OSC 110995 | DQ522550 | DQ518764 | DQ522379 | DQ522430 | DQ522334 |
|  | *Ophiocordyceps rhizoidea* | NHJ 12529 | EF468969 | EF468824 | EF468872 | EF468922 | EF468765 |
|  | *Ophiocordyceps rhizoidea* | NHJ 12522 | EF468970 | EF468825 | EF468873 | EF468923 | EF468764 |
|  | *Ophiocordyceps ryogamiensis* | NBRC 101751 | KF049614 | KF049633 | KF049650 |  | KF049688 |
|  | *Ophiocordyceps sinensis* | CO18 |  |  |  |  |  |
|  | *Ophiocordyceps sinensis* | EFCC 7287 | EF468971 | EF468827 | EF468874 | EF468924 | EF468767 |
|  | *Ophiocordyceps sinensis* (*Hirsutella sinensis*) | CGB999335 | KU239984 | KU239985 | KU239986 | KU239987 | KU239989 |
|  | *Ophiocordyceps sobolifera* | TNS F18521 | KJ878933 | KJ878898 | KJ879013 |  | KJ878979 |
|  | *Ophiocordyceps sobolifera* | KEW 78842 | EF468972 | EF468828 | EF468875 | EF468925 |  |
|  | *Ophiocordyceps sp.* | TNS F18495 | KJ878934 | KJ878901 | KJ879017 |  |  |
|  | *Ophiocordyceps sp.* | OSC 110997 | EF468976 |  | EF468879 | EF468929 | EF468774 |
|  | *Ophiocordyceps sp.* | OSC 151904 | KJ878935 | KJ878899 | KJ879014 |  | KJ878980 |
|  | *Ophiocordyceps sp.* | OSC 151905 | KJ878936 |  | KJ879015 | KJ878951 | KJ878982 |
|  | *Ophiocordyceps sp.* | OSC 151909 | KJ878937 | KJ878900 | KJ879016 | KJ878952 | KJ878981 |
|  | *Ophiocordyceps sphecocephala* | OSC 110998 | DQ522551 | DQ518765 | DQ522381 | DQ522432 | DQ522336 |
|  | *Ophiocordyceps stylophora* | OSC 110999 | EF468982 | EF468837 | EF468882 | EF468931 | EF468777 |
|  | *Ophiocordyceps stylophora* | OSC 111000 | DQ522552 | DQ518766 | DQ522382 | DQ522433 | DQ522337 |
|  | *Ophiocordyceps tricentri* | NBRC 106968 | AB968393 | AB968423 |  | AB968554 | AB968593 |
|  | *Ophiocordyceps unilateralis* | OSC 128574 | DQ522554 | DQ518768 | DQ522385 | DQ522436 | DQ522339 |
|  | *Ophiocordyceps variabilis* | OSC 111003 | EF468985 | EF468839 | EF468885 | EF468933 | EF468779 |
|  | *Ophiocordyceps yakusimensis* | HMAS_199604 | KJ878938 | KJ878902 | KJ879018 | KJ878953 |  |
|  | *Paecilomyces lilacinus* | ARSEF 2181 | AF339583 | AF339534 | EF468896 |  | EF468790 |
|  | *Paecilomyces lilacinus* | CBS 284.36 | AY624189 | FR775484 | EF468898 | EF468941 | EF468792 |
|  | *Paecilomyces lilacinus* | CBS 431.87 | AY624188 | EF468844 | EF468897 | EF468940 | EF468791 |
|  | *Polycephalomyces cuboideus* | NBRC 101740 | KF049610 | KF049629 | KF049646 |  | KF049684 |
|  | *Polycephalomyces formosus* | ARSEF 1424 | KF049615 | AY259544 | DQ127245 | KF049671 | DQ118754 |
|  | *Polycephalomyces nipponicus* | BCC 1881 | KF049618 | KF049636 |  | KF049674 | KF049692 |
|  | *Polycephalomyces nipponicus* | BCC 1682 | KF049620 | KF049638 |  |  | KF049694 |
|  | *Polycephalomyces nipponicus* | NHJ 4268 | KF049621 | KF049639 | KF049654 | KF049676 | KF049695 |
|  | *Polycephalomyces nipponicus* | BCC 18108 | KF049608 | KF049626 | KF049644 |  | KF049681 |
|  | *Polycephalomyces nipponicus* | BCC 2325 | KF049622 | KF049640 | KF049655 | KF049677 | KF049696 |
|  | *Polycephalomyces paracuboideus* | NBRC 101742 | KF049611 | KF049630 | KF049647 | KF049669 | KF049685 |
|  | *Polycephalomyces prolificus* | TNS-F-18481 | KF049612 | KF049631 | KF049648 |  | KF049686 |
|  | *Polycephalomyces prolificus* | TNS-F-18547 | KF049613 | KF049632 | KF049649 | KF049670 | KF049687 |
|  | *Polycephalomyces ramosopulvinatus* | SU-65 |  | DQ118742 | DQ127244 |  | DQ118753 |
|  | *Polycephalomyces ramosopulvinatus* | EFCC 5566 |  | KF049627 | KF049645 |  | KF049682 |
|  | *Polycephalomyces sp.* | JB07.08.16_08 | KF049616 | KF049635 | KF049652 | KF049672 | KF049690 |
|  | *Polycephalomyces sp.* | JB07.08.17_07b | KF049617 |  | KF049653 | KF049673 | KF049691 |
|  | *Polycephalomyces sp.* | BBC 2637 | KF049619 | KF049637 |  | KF049675 | KF049693 |
|  | *Polycephalomyces tomentosus* | BL 4 | KF049623 | KF049641 | KF049656 | KF049678 | KF049697 |
|  | *Stilbella buquetii* | HMAS_199617 | KJ878940 | KJ878905 | KJ879020 |  | KJ878985 |
|  | *Stilbella buquetii* | HMAS_199613 | KJ878939 | KJ878904 | KJ879019 |  | KJ878984 |
|  | *Tilachlidiopsis nigra* | TNS 16250 | KJ878942 |  | KJ879021 |  | KJ878987 |
|  | *Tilachlidiopsis nigra* | TNS 16252 | KJ878941 | KJ878906 |  |  | KJ878986 |
|  | *Tolypocladium cylindrosporum* | NRRL 28025 | AF049153 | AF049173 |  |  |  |
